# TRIM24 controls induction of latent HIV-1 by stimulating transcriptional elongation

**DOI:** 10.1038/s42003-023-04484-z

**Published:** 2023-01-23

**Authors:** Riley M. Horvath, Matthew Dahabieh, Tom Malcolm, Ivan Sadowski

**Affiliations:** Department of Biochemistry and Molecular Biology, Molecular Epigenetics Group, LSI, University of British Columbia, Vancouver, B.C. Canada

**Keywords:** Transcriptional regulatory elements, Virology

## Abstract

Binding of USF1/2 and TFII-I (RBF-2) at conserved sites flanking the HIV-1 LTR enhancer is essential for reactivation from latency in T cells, with TFII-I knockdown rendering the provirus insensitive to T cell signaling. We identified an interaction of TFII-I with the tripartite motif protein TRIM24, and these factors were found to be constitutively associated with the HIV-1 LTR. Similar to the effect of TFII-I depletion, loss of TRIM24 impaired reactivation of HIV-1 in response to T cell signaling. TRIM24 deficiency did not affect recruitment of RNA Pol II to the LTR promoter, but inhibited transcriptional elongation, an effect that was associated with decreased RNA Pol II CTD S2 phosphorylation and impaired recruitment of CDK9. A considerable number of genomic loci are co-occupied by TRIM24/TFII-I, and we found that *TRIM24* deletion caused altered T cell immune response, an effect that is facilitated by TFII-I. These results demonstrate a role of TRIM24 for regulation of transcriptional elongation from the HIV-1 promoter, through its interaction with TFII-I, and by recruitment of P-TEFb. Furthermore, these factors co-regulate a significant proportion of genes involved in T cell immune response, consistent with tight coupling of HIV-1 transcriptional activation and T cell signaling.

## Abstract (web summary)

TRIM24 regulates transcriptional elongation from the HIV-1 promoter through its interaction with TFII-I, and by recruitment of P-TEFb.

## Introduction

Advances in antiretroviral therapy (ART) has eliminated HIV/AIDS as a leading cause of death globally, but this treatment does not represent a cure because of the long-lived reservoir of latently infected cells which develop in infected people, that is unaffected by ART^1–3^. Various strategies to eliminate latently infected cells involving modulation of provirus expression by latency-reversing (LRAs) or latency-promoting agents (LPAs), are currently under investigation^4,5^. Expression of HIV-1 from the 5’ long terminal repeat (LTR)^6,7^ is tightly linked to T-cell activation, through the function of factors that bind the LTR enhancer region, regulated by T-cell signaling^8^. The Ras-response elements (RBE3 and RBE1), located flanking the LTR enhancer region, were identified as required for response to activated Ras signaling^9^, and represent the most highly conserved LTR *cis-*elements in provirus from patients that develop AIDS^10^. These conserved elements bind Ras-response element binding factor 2 (RBF-2), comprised of Upstream Stimulatory Factor 1 and 2 (USF1/2) and TFII-I (*GTF2I*)^11,12^. Mutation of the RBE1 and RBE3 elements inhibit induction of HIV-1 in response to T-cell activation^12–14^, although the mechanism for this effect has not been elucidated. RBF-2 is also associated with YY1 at the upstream RBE3 element^15^, and these factors may mutually regulate binding to this region of the LTR^13^. However, unlike TFII-I, USF1/2 which are constitutively bound to the LTR, YY1 is dissociated in response to T-cell activation but is bound to the LTR of provirus that produce immediate latency^15^.

TFII-I, encoded by *GTF2I*, exerts negative or positive effects on transcription, dependent upon promoter context and cell differentiation state^16^. TFII-I plays a vital role for RBF-2 formation and function, as USF1/2 has low affinity for RBE3/1 in its absence in vitro^12,17^. TFII-I was initially identified bound to the adenovirus major late (AdML) core promoter, as well as transcriptional initiator (Inr) elements^18^, but was subsequently observed associated with sequence-specific transcription factors, including SRF, STAT1/3, ATF6, and USF1/2^16^. TFII-I contributes to transcriptional activation, but was also associated with repression through the recruitment of HDAC3 and the PRC2 component SUZ12^19^. Accordingly, mutation of the RBE1/3 elements inhibits reactivation of latent HIV-1 in response to T-cell activation, but also cause elevated basal expression of LTR reporter genes^12,14^. These observations indicate a requirement of TFII-I for regulation of HIV-1 transcription, but the precise mechanistic role of its effect on viral transcription has not been determined.

Tripartite-Motif protein 24 (TRIM24), previously designated TIF1α, possesses amino-terminal RBCC (RING, BBox, coiled-coil) domains in addition to a Transcription Intermediary Family (TIF1)-defining carboxy-terminal tandem plant homeodomain-bromodomain (PHD-BRD) motif^20^. TRIM24 is associated with both oncogenic and tumor suppressive effects; deletion of *TRIM24* promotes hepatocellular carcinoma in mice, while overexpression negatively correlates with cancer progression, including breast and prostate cancers^21–23^. Although mechanisms for the development and progression of these cancers are ill-defined, TRIM24-dependent transcriptional regulation is likely involved. Specifically, TRIM24 was characterized as a cofactor of various nuclear receptors, including for estrogen (ER) and androgen (AR), and it was proposed that recruitment of TRIM24 to genes regulated by these factors promotes cellular proliferation and tumor growth^21,23–25^. In addition to transcription factor-mediated recruitment, TRIM24 interacts with H3K4me0/K23ac modified histones through its tandem PHD-BRD domains^21,23,26^, and this promotes its SUMOylation, which causes global alterations in transcription^27^, but overall, the molecular mechanism(s) by which TRIM24 functions as a coactivator of transcription has not been established.

Here, we demonstrate that TRIM24 directly interacts with TFII-I, facilitating its recruitment to the HIV-1 LTR. The cofactor TRIM24 promotes transcriptional elongation from the viral promoter in response to T-cell signaling through enhanced recruitment of CDK9 and elevated Ser-2 phosphorylation of the C-terminal domain of RNAPII. Furthermore, TRIM24 and TFII-I display considerable genome-wide co-occupancy and regulate a significant proportion of genes involved in cellular adhesion for T-cell activation response. These observations provide mechanistic insight into the function of TFII-I and regulation of HIV-1 transcription for control of reactivation from provirus latency and reveal a mechanism for TRIM24 as a transcriptional coactivator for transcriptional elongation.

## Results

### TFII-I is required for reactivation of HIV-1 provirus

**Fig. 1:**
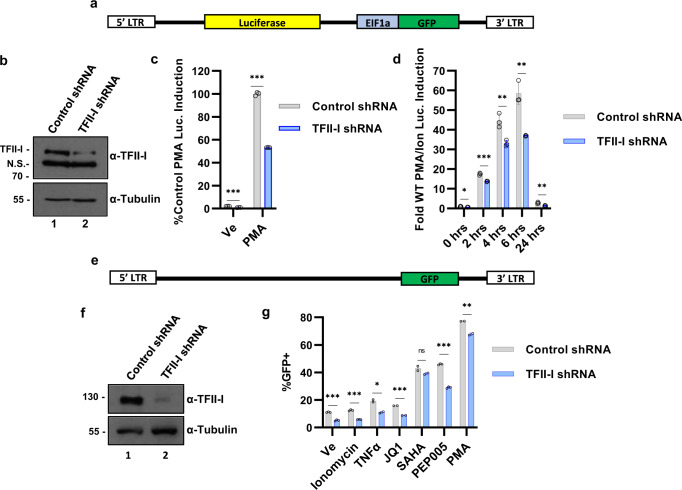
TFII-I promotes HIV-1 expression. **a** Schematic representation of the reporter virus integrated into the Jurkat Tat mHIV-Luciferase cell line, where luciferase is expressed from the 5’ LTR as a fusion with p24^gag^. **b** Jurkat mHIV-Luciferase cells were transduced with pLKO shRNA vector control (lane 1) or TFII-I shRNA expressing lentivirus (lane 2). Immunoblots were performed on whole-cell lysates prepared 8 days post puromycin selection, with antibodies against TFII-I or Tubulin. **c** Jurkat mHIV-Luciferase cells transduced with control shRNA or TFII-I shRNA expression vectors were left untreated (Ve, DMSO) or stimulated with 20 nM PMA for 4 h prior to measuring luciferase activity (*n* = 3, mean ± SD). **d** Transduced Jurkat mHIV-Luciferase cells were treated with 20 nM PMA/1 µM ionomycin for the indicated time prior to measuring luciferase activity (*n* = 3, mean ± SD). **e** Schematic representation of the Jurkat-based JLat10.6 HIV-1 proviral cell model, where *Nef* is replaced with GFP and the virus is rendered replication incompetent by a mutation within *Env*. **f** JLat10.6 cells were transduced with empty Control shRNA vector (lane 1) or TFII-I targeting shRNA (lane 2). Following selection with puromycin for 4 days, whole-cell lysates were collected and analyzed by western blot using antibodies against TFII-I or Tubulin. **g** Four days later—shRNA infection-transduced JLat10.6 cells were treated with DMSO (Ve), 1 µM ionomycin, 10 ng/µL TNFα, 10 µM JQ1, 10 µM SAHA, 10 nM PEP005, or 10 nM PMA. Following 20 h incubation, cells were analyzed by flow cytometry (*n* = 2, mean ± SD).

Mutation of the RBE3/1 binding sites for RBF-2/TFII-I prevents induction of HIV-1 in response to T-cell signaling^12,17^. These *cis*-elements are also necessary for binding of USF1/2, and YY1^15^, and so to examine the effect of TFII-I in isolation on LTR-directed transcription, we knocked down its expression using shRNA in the Jurkat mHIV-Luciferase line where luciferase is expressed from the HIV-1 5’ LTR as a fusion with p24^gag^ (Fig. 1a, b)^28^. We observed that knockdown of TFII-I impaired expression of luciferase in these cells treated with PMA (Fig. 1c), or a combination of PMA and ionomycin (Fig. 1d), treatments which induce signaling pathways stimulated by T-cell activation^29^. Similar results were observed with the knockdown of TRIM24 by shRNA in JLat10.6 cells, which bears a full-length HIV-1 provirus in which *Nef* is substituted for GFP^30^ (Fig. 1e, f). In the JLat10.6 cell line, we observed that TFII-I depletion inhibited reactivation of provirus expression in response to a variety of previously characterized LRAs, with the notable exception of the histone deacetylase inhibitor (HDACi) suberanilohydroxamic acid (SAHA) (Fig. 1g). In addition, we examined whether T-cell activation affected expression of TFII-I or TRIM24 and found that PMA/ionomycin treatment did not alter protein levels (Supplementary Fig. 1a) although a slight increase in *GTF2I* (TFII-I) mRNA abundance was detected (Supplementary Fig. 1b), but no effect on TRIM24 mRNA (Supplementary Fig. 1c). Consequently, activation of HIV-1 transcription in response to T-cell signaling likely does not involve the elevated expression of TFII-I or TRIM24 protein^13^. Collectively, these results support previous observations showing that mutation of the RBE *cis*-elements, which bind TFII-I, impairs response of the 5’ LTR to T-cell activation signals^12–14,17^.

### TFII-I interacts with TRIM24

**Fig. 2:**
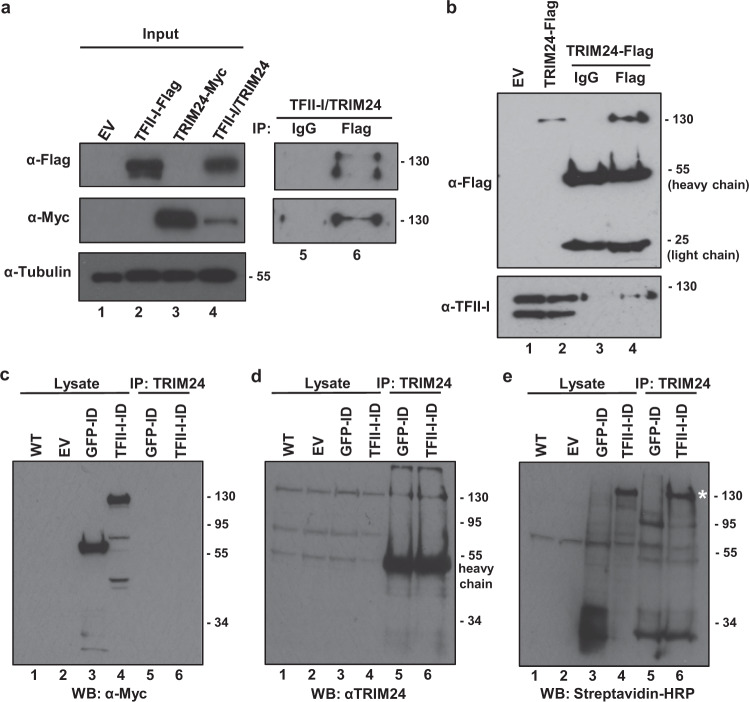
TRIM24 interacts with TFII-I. **a** HEK293T cells were transfected with an empty vector or plasmids expressing TFII-I-Flag or TRIM24-Myc (lanes 1–4). Lysates were analyzed by immunoblotting with antibodies against the Flag, Myc, or Tubulin as indicated. Cells co-expressing TFII-I-Flag and TRIM24-Myc were immunoprecipitated with control (IgG) (lane 5) or Flag (lane 6) antibodies and complexes were analyzed by immunoblotting with Flag and Myc antibodies as indicated. **b** Lysates from Jurkat cells bearing a vector control (EV, lane 1) or a TRIM24-Flag expression vector (lane 2) were analyzed by immunoblotting with antibodies against Flag or TFII-I. Jurkat cells expressing TRIM24-Flag were immunoprecipitated with control (IgG, lane 3), or anti-Flag (lane 4) antibodies, and complexes were analyzed by immunoblotting with anti-Flag or anti-TFII-I antibodies as indicated. **c**–**e** HEK293T (lane 1) or cells expressing GFP-TurboID-Myc (lanes 3 and 5), TFII-I-TurboID-Myc (lanes 4 and 6), or an empty vector (lane 2) were incubated with 500 µM biotin for 1 h. Cell lysates (lanes 1–4) or TRIM24 immunoprecipitants (lanes 5-6) were analyzed by immunoblotting with antibodies against Myc (**c**), TRIM24 (**d**), or with Streptavidin-HRP (**e**). Biotinylated TRIM24 is indicated with a white asterisk (**e**, lane 6, ~130 kDa).

To characterize mechanism(s) for regulation of HIV-1 transcription by TFII-I, we looked for novel interacting proteins using a 2-hybrid screen modified for use with transcriptional activator proteins^31^. Using a GAL4-TFII-I bait we isolated multiple clones encoding TUP1-TRIM24 fusions from a human T-cell cDNA library. To determine whether this interaction identified in yeast can be detected in human cells, we co-transfected HEK293T cells with plasmids expressing epitope-tagged TFII-I and TRIM24, where we observe TRIM24-Myc associated with TFII-I-Flag immunoprecipitated from cell lysates (Fig. 2a, lane 6). We performed a similar experiment in the Jurkat T-cell line where we expressed TRIM24-Flag and observed interaction with endogenous TFII-I by co-immunoprecipitation (Fig. 2b, lane 4).

We also examined interaction of TFII-I and TRIM24 in HEK293T cells using proximity labeling with Bio-ID, where we transfected HEK293T cells with plasmids expressing TFII-I or GFP as a fusion with BirA^*^ (TurboID) and Myc epitope tags (Fig. 2c, d and Supplementary Fig. 2a). Upon biotin addition, we observed that the TFII-I and GFP-TurboID fusions cause biotinylation of cellular proteins within 1 h, and this effect persisted up to 24 h (Supplementary Fig. 2b). Importantly, we find that TRIM24 becomes biotinylated in cells expressing the TFII-I-BirA* fusion (Fig. 2e, lane 6), but not in cells expressing GFP-BirA* (Fig. 2e, lane 5). Proximity labeling of TRIM24 by TFII-I-BirA* indicates that these factors associate in the context of living cells.

### TRIM24 activates expression from the HIV-1 LTR

**Fig. 3:**
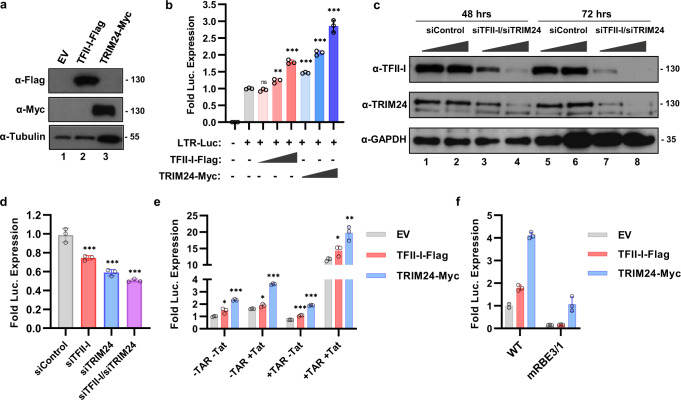
HIV-1 LTR transcription is activated by TRIM24. **a** HEK293T cells were transfected with plasmids expressing TFII-I-Flag (lane 2), TRIM24-Myc (lane 3), or an empty vector (EV, lane 1). Lysates were analyzed by immunoblotting against Myc, Flag, or Tubulin as indicated. **b** HEK293T cells co-transfected with an LTR-Luciferase reporter plasmid and TFII-I-Flag or TRIM24-Myc expression vectors were analyzed for luciferase activity (*n* = 3, mean ± SD). **c** HEK293T cells were transfected with siRNA against TFII-I or TRIM24 (lanes 3-4 and lanes 7-8). Lysates were collected 2- (lanes 1–4) and 3 days (lanes 5–8) post transfection and analyzed by immunoblotting with anti-TRIM24, anti-TFII-I, or anti-GAPDH antibodies as indicated. Loading control was performed on an individual gel. **d** HEK293T cells co-transfected with an LTR-Luciferase reporter plasmid and siRNA targeting TFII-I and/or TRIM24 were analyzed for luciferase expression (*n* = 3, mean ± SD). **e** Luciferase assay was performed using HEK293T cells co-transfected with an LTR-Luciferase reporter plasmid and TRIM24-Myc and/or TFII-I-Flag expression vector, in the presence (+ Tat) or absence (−Tat) of Tat expression vector. The LTR-Luciferase reporter contained (+TAR) or lacked the TAR (−TAR) stem loop region (*n* = 3, mean ± SD). **f** Wild-type LTR-Luciferase reporter or LTR-Luciferase reporter with mutated RBE3 and RBE1 elements (mRBE3/1) were co-transfected with an empty vector plasmid (EV) or plasmids expressing TFII-I-Flag or TRIM24-Myc (*n* = 3, mean ± SD).

Because TRIM24 interacts with TFII-I in both HEK293T and Jurkat T cells, we examined whether this factor affected expression from the HIV-1 LTR. To this end, we co-transfected HEK293T cells with an LTR-Luciferase reporter construct and increasing amounts of plasmids expressing TRIM24-Myc or TFII-I-Flag (Fig. 3a). Here, we observed a dose-dependent, threefold increase in transcriptional activity of the LTR-Luciferase reporter upon TRIM24 overexpression relative to endogenous levels (Fig. 3b). In contrast, we observed only a modest 1.7-fold increase in luciferase expression when TFII-I-Flag was overexpressed (Fig. 3b). These data are consistent with the previously described role of TFII-I at the HIV-1 LTR^12,17^, and suggests that TRIM24 represents a required cofactor for transcriptional activation.

To further examine the role of TRIM24 in the activation of HIV-1 expression, we determined the effects of TRIM24 and TFII-I knockdown. First, we validated siRNA SmartPools against TRIM24 and TFII-I in transiently transfected HEK293T cells. Both pools were efficient at knocking down expression as determined by immunoblotting, within 48 h post transfection; however, knockdown was more pronounced at 72 h (Fig. 3c). We then co-transfected siRNA pools with LTR-Luciferase reporter constructs into HEK293T cells, and measured luciferase activity 72 h later. Here, we observed a ~40% decrease in luciferase activity in TRIM24 knockdown cells, relative to the control (Fig. 3d). Consistent with results from overexpression, knockdown of TFII-I resulted in a more modest ~25% decrease in luciferase activity, and knockdown of both TFII-I and TRIM24 produced an effect similar to knockdown of TRIM24 alone (Fig. 3d).

We also assessed the effect of TRIM24 for Tat-dependent activation of the HIV-1 LTR by transfected HEK293T cells with LTR-Luciferase reporter constructs either bearing or lacking the Tat-responsive element TAR, in the presence or absence of a plasmid expressing the viral transactivator Tat^17^. Regardless of the presence of TAR and/or Tat, overexpression of TRIM24 and TFII-I resulted in elevated expression of luciferase (Fig. 3e). As such, the effect of TRIM24 on LTR-directed expression is largely independent of Tat function.

Finally, we analyzed the effect of TFII-I or TRIM24 overexpression on the induction of an LTR-Luciferase construct bearing RBE3/1 elements with mutations known to abrogate RBF-2 (TFII-I) binding^17^. We found that TFII-I-Flag transfection did not induce luciferase expression from an HIV-1 LTR reporter in which the RBE elements were mutated (Fig. 3f). Upon transfection of TRIM24-Myc, we observed a slight increase in luciferase expression from the mRBE3/1 LTR reporter, however, the observed luciferase induction was far less than for that of the wild-type LTR (Fig. 3f). These results confirm that TFII-I and TRIM24 require intact RBE3 and RBE1 elements for full induction of HIV-1 LTR transcription.

### TRIM24 is required for induction of HIV-1 provirus expression

**Fig. 4:**
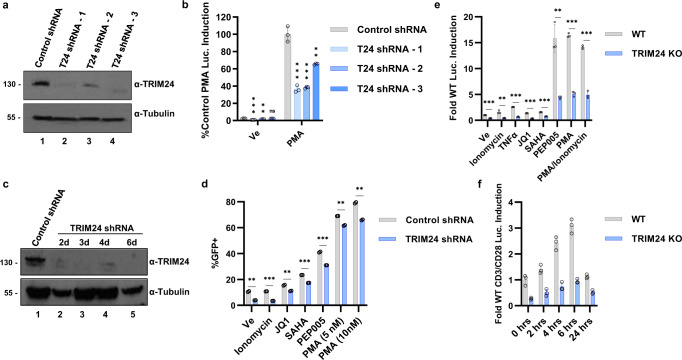
TRIM24 is an activator of HIV-1 expression in T cells. **a** Jurkat mHIV-Luciferase cells were transduced with pLKO empty vector control (lane 1) or various TRIM24 targeting shRNA (lanes 2–4). Following 3 days of puromycin selection, lysates from transduced cells were analyzed by immunoblotting with antibodies against TRIM24 or Tubulin. **b** Jurkat mHIV-Luciferase cells transduced with control shRNA vector or TRIM24 targeting shRNA were left untreated (Ve, DMSO) or stimulated with 20 nM PMA for 4 h prior to measuring luciferase activity (*n* = 3, mean ± SD). **c** JLat10.6 cells were transduced with a Control shRNA vector or a TRIM24 targeting shRNA. Following puromycin selection, whole-cell lysates were extracted and subject to immunoblotting against TRIM24 or Tubulin. **d** Control shRNA or TRIM24 targeting shRNA transduced JLat10.6 cells were cultured for 4 days with puromycin and subsequently incubated with DMSO (Ve), 1 µM ionomycin, 10 µM JQ1, 10 µM SAHA, 10 nM PEP005, and 5 nM or 10 nM PMA, cells were examined 20 h later by flow cytometry (*n* = 2, mean ± SD). **e** Wild-type or *TRIM24* KO Jurkat mHIV-Luciferase cells were incubated with DMSO (Ve), 1 µM ionomycin, 10 ng/µL TNFα, 10 µM JQ1, 10 µM SAHA, 10 nM PEP005, 10 nM PMA or 10 nM PMA/1 µM ionomycin for 4 h prior to measuring luciferase expression (*n* = 3, mean ± SD). **f** Wild-type or *TRIM24* KO Jurkat mHIV-Luciferase were cultured in the presence of CD3/CD28 coated beads with luciferase expression analyzed at the indicated time points (*n* = 3, mean ± SD).

The results shown above indicate that TRIM24 interacts with TFII-I, and its presence correlates with the activation of HIV-1 LTR-directed transcription in HEK293T cells. We next examined the requirement of this factor for HIV-1 provirus expression in T cells. We found that T-cell activation in response to PMA/ionomycin treatment did not alter TRIM24 expression (Supplementary Fig. 1a, c). Next, we used shRNA to knockdown TRIM24 expression (Fig. 4a) in the Jurkat mHIV-Luciferase cell line where luciferase expression is driven by the 5’ LTR^14^ (Fig. 1a). For all shRNAs used, we observed a substantial decrease in luciferase expression in response to PMA compared to the non-targeting control shRNA vector (Fig. 4b). Similarly, we also performed TRIM24 shRNA knockdown in JLat10.6 cells (Fig. 4c) and observed that TRIM24 depletion inhibited latency reversal in response to all agonists and latency-reversing agents used, as measured by the percent of cells that expressed GFP (Fig. 4d).

We also examined the effect of TRIM24 on HIV-1 expression using CRISPR-Cas9-mediated gene disruption in the Jurkat mHIV-Luciferase cell line (Supplementary Fig. 3a). Consistent with the results with shRNAs, each *TRIM24* knockout (TRIM24 KO) clonal cell line displayed a significant reduction in HIV-1 expression in response to treatment with PMA, as measured by luciferase activity (Supplementary Fig. 3b). Furthermore, induction of HIV-1 expression in response to treatment with various latency-reversing agents (LRAs) was significantly inhibited in the *TRIM24* knockout lines (Fig. 4e). In addition, we observed that *TRIM24* KO cells produced significantly impaired HIV-1 reactivation in response to T-cell activation as induced by CD3/CD28 mediated T-cell receptor cross-linking (Fig. 4f).

**Fig. 5:**
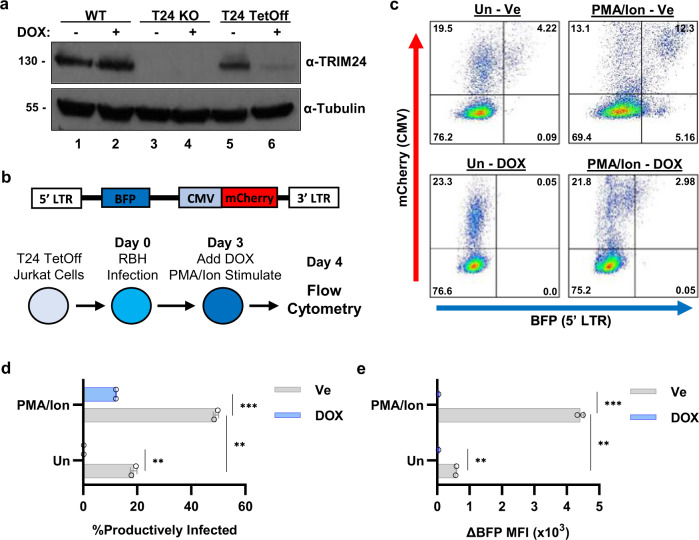
*TRIM24* knockout encourages the establishment of immediate latency. **a** Wild-type, *TRIM24* KO, or TRIM24 TetOff Jurkat cells were untreated or incubated with 1 µg/mL doxycycline for 20 h, when whole-cell lysates were analyzed by immunoblotting with TRIM24 or Tubulin antibodies. **b** Schematic depiction of the Red-Blue-HIV-1 reporter virus (RBH) assay. *TRIM24* knockout Jurkat cells transduced with a TetOff TRIM24 expression lentivirus were infected with RBH. Three days post infection, cells were untreated or incubated with 1 µg/mL doxycycline (DOX); 2 h post DOX treatment, cells were treated with a vehicle control (DMSO) or 10 nM PMA/1 µM ionomycin. Flow cytometry was performed 20 h later to measure infection (red) and HIV-1 (blue) reporter expression. **c** Representative flow cytometric scatterplots of cells treated as described in (**b**); mCherry intensity is displayed on the *Y* axis, and BFP expression on the *X* axis. For each scatterplot, the bottom left quadrant indicates uninfected cells (mCherry−/BFP−), the top left indicates the latent population(mCherry+/BFP−), the top right indicates productively infected cells (mCherry +/BFP+), and the bottom right depicts noise generated from viral rearrangements (BFP+). **d** Summary of scatterplot data produced from RBH TRIM24 rescue experiments. Shown is the percentage of productively infected cells, with error bars representing standard deviation (*n* = 2, mean ± SD). **e** Same as (**d**), but data are presented as the change in BFP mean fluorescent intensity (MFI) as compared to RBH uninfected, fluorescent-negative cells (*n* = 2, mean ± SD).

To confirm that the effects on HIV-1 expression observed in the *TRIM24* knockout cells is not caused by an indirect effect, we generated a Jurkat T-cell line in which endogenous *TRIM24* is deleted, and the factor is complemented by conditional expression of TRIM24 under the control of a tetracycline repressed promoter (Fig. 5a). The TRIM24 TetOff cell line was infected with a modified Red-Blue dual HIV-1 reporter derivative which expresses BFP from the 5’ LTR and mCherry from an internal CMV promoter to enable detection of infected cells independently from LTR-directed transcription (RBH, Fig. 5b)^32^. Three days post infection with RBH we depleted TRIM24 by addition of doxycycline, and cells were left untreated, or stimulated with PMA and ionomycin; the infected cells were analyzed for latently and productively infected cells the following day by flow cytometry (Fig. 5b). This analysis found that depletion of TRIM24 caused a dramatic reduction in the proportion of productively infected cells, as indicated by cells expressing CMV-mCherry and BFP from the LTR, in both the untreated and PMA/ionomycin stimulated populations compared to cells expressing TRIM24 (Fig. 5c). In untreated cells we found that TRIM24 depletion caused near complete establishment of latent provirus, and in PMA/ionomycin-treated cells we observed a reduction in productively infected cells to ~10% from the normal proportion of ~50% (Fig. 5d). Furthermore, in both untreated and PMA/ionomycin-treated cells, we observed a significant reduction in levels of 5’ LTR driven transcription upon TRIM24 depletion as determined by BFP mean fluorescence intensity (MFI) of infected cells (Fig. 5e). Collectively, these results indicate that TRIM24 is essential for full induction of HIV-1 expression in response to T-cell activation signals as well as many LRAs.

### Interaction of TRIM24 with the HIV-1 LTR requires TFII-I

**Fig. 6:**
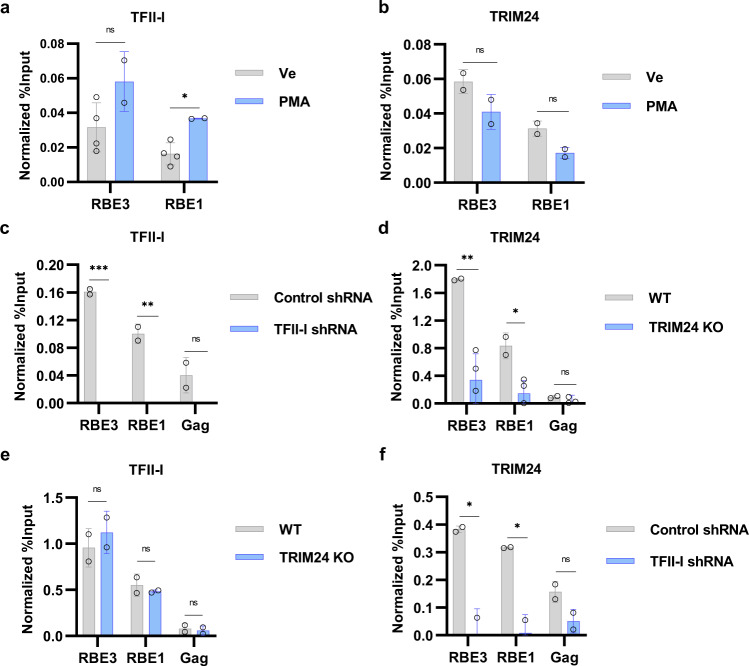
TFII-I/TRIM24 co-localize to the HIV-1 LTR. **a**, **b** Jurkat Tat mdHIV Clone 11 cells were left untreated (Ve, DMSO) or incubated with 50 nM PMA for 24 h. ChIP-qPCR was performed using anti-TFII-I (**a**) or anti-TRIM24 (**b**) antibodies. **c** ChIP-qPCR analysis was performed using TFII-I antibodies with Jurkat mHIV-Luciferase cells transduced with pLKO lentiviral control shRNA or TFII-I targeting shRNA. **d** As in (**c**) but anti-TRIM24 antibodies were used for ChIP analysis. **e** Wild-type or *TRIM24* KO Jurkat mHIV-Luciferase cells were subject to ChIP-qPCR analysis using anti-TRIM24 antibodies. **f** As in (**e**) but anti-TFII-I antibodies were used for ChIP analysis. ChIP-qPCR results are normalized by subtraction of values produced with sample-paired non-specific IgG (*n* = 2–4, mean ± SD).

To further assess the relationship between TFII-I and TRIM24 for regulation of HIV-1 expression, we examined their occupancy on the HIV-1 LTR using ChIP-qPCR. Consistent with previous observations^15^, we observe interaction of TFII-I at the LTR regions representing both RBE3 and RBE1 in the presence or absence of PMA stimulation (Fig. 6a), and a similar result was observed for TRIM24 (Fig. 6b); however, we do observe recruitment of NFκB p65 to the enhancer region in response to PMA treatment of these same cells (Supplementary Fig. 4). These observations indicate that, consistent with the interaction as measured by co-IP and Bio-ID (Fig. 2), TRIM24 co-localizes with TFII-I on the HIV-1 LTR in T cells. Similarly, the association of TFII-I and TRIM24 was observed with the HIV-1 LTR in the HeLa-derived TZM-bl cell line, in both unstimulated and PMA-treated cells (Supplementary Fig. 5).

To examine the requirement of TFII-I for recruitment of TRIM24 to the LTR and vice versa, we performed ChIP-qPCR in cells where these factors were depleted. We first confirmed that depletion of TFII-I and TRIM24 resulted in the loss of their respective ChIP signals as measured with primers specific for the HIV-1 LTR (Fig. 6c, d). We found that association of TFII-I with the LTR was unaffected by *TRIM24* KO (Fig. 6e). In contrast, interaction of TRIM24 with both the RBE3 and RBE1 regions was significantly reduced in cells where TFII-I expression was knocked down with shRNA (Fig. 6f). Thus, loss of TRIM24 has no effect on binding of TFII-I to its sites on the LTR but the interaction of TRIM24 with the LTR is dependent upon TFII-I. These observations support the contention that TFII-I directly recruits TRIM24 to the HIV-1 LTR.

### TRIM24 stimulates HIV-1 transcriptional elongation

**Fig. 7:**
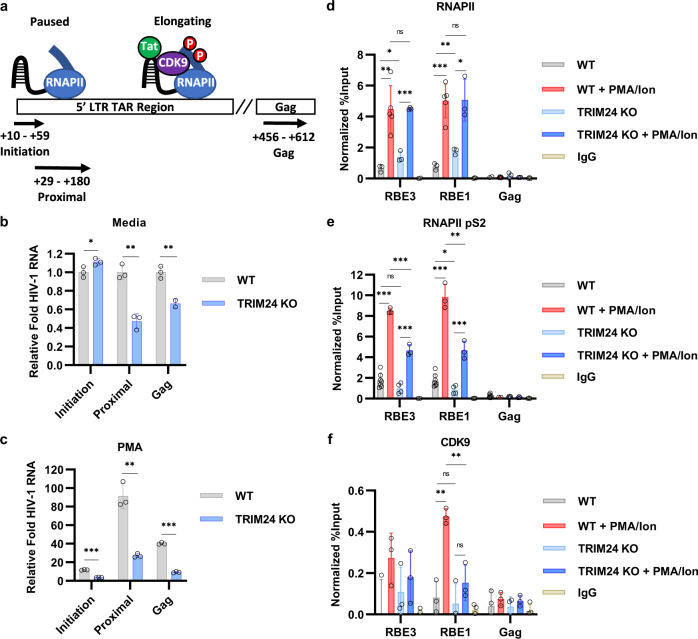
TRIM24 stimulates elongation of HIV-1 transcription. **a** Schematic representation of the 5’ LTR TAR and Gag-encoding regions, indicating primer pairs used to measure Initiation (+10–59 bp), Proximal (+29–180 bp), or Gag (+456–612 bp) HIV-1 transcript abundance. **b**, **c** Wild-type and *TRIM24* KO Jurkat mHIV-Luciferase cells were cultured under normal conditions (**b**) or with 20 nM PMA for 4 h (**c**). RNA was extracted, cDNA synthesized, and RT-PCR was performed using primers to detect Initiation, Proximal and Gag RNA sequences with data normalized to the untreated wild-type control (*n* = 3, mean ± SD). **d**–**f** Wild-type or *TRIM24* KO Jurkat mHIV-Luciferase cells were left untreated or stimulated with 20 nM PMA/1 μM ionomycin for 4 h. ChIP-qPCR analysis was performed using anti-RNAPII (**d**), anti-RNAPII pS2 (**e**), or anti-CDK9 (**f**) antibodies. Results were normalized by subtraction of values produced with sample-paired non-specific IgG (*n* = 3–7, mean ± SD).

We examined the mechanism for regulation of HIV-1 transcription by TRIM24 by measuring the effect of this factor on the initiation and elongation of RNAPII from the LTR promoter. For this, we employed an RT-PCR assay for mRNAs representing initiating versus elongating proximal and Gag transcripts (Fig. 7a)^33,34^. In these experiments, we observed a small increase in the abundance of HIV-1 RNA associated with paused RNA Polymerase II upon TRIM24 depletion as compared to wild-type in untreated cells (Fig. 7b). In contrast, *TRIM24* KO was associated with a marked decrease in both proximal and Gag elongating mRNA transcripts relative to wild-type under basal conditions (Fig. 7b). Similarly, in cells stimulated with PMA, we observe significant reduction in promoter-proximal and *gag* elongated transcripts, and to a lesser extent, initiation transcripts, in *TRIM24* KO cells relative to WT (Fig. 7c). Taken together, these results suggest that loss of TRIM24 function causes a defect in transcriptional elongation from the HIV-1 LTR. We note that this assay is complicated by the fact that all HIV-1 transcripts possess the same first 69 nucleotides of viral mRNA (Fig. 7a), and consequently the difference in initiation transcripts between *TRIM24* KO and wild-type cells is exaggerated in PMA-treated cells because of accumulation of elongated mRNAs.

To further examine the effect of TRIM24 on HIV-1 transcription, we measured its role in the recruitment and modification of RNAPII using ChIP-qPCR. Enhanced interaction of RNAPII with the LTR was observed in cells treated with PMA and ionomycin, relative to untreated cells (Fig. 7d). In *TRIM24* KO cells, we observed a slight increase in RNAPII occupancy at the LTR promoter in untreated cells and no difference in the recruitment of RNAPII to the LTR upon PMA/ionomycin mediated T-cell activation as compared to WT cells (Fig. 7d). These findings indicate that TRIM24 is dispensable for initial recruitment of RNAPII to the HIV-1 promoter. Of note, consistent with previous studies^35–38^ we observed minimal enrichment of RNAP at the proviral Gag region, compared to the LTR, an effect that is likely a consequence of high turnover rate of RNA Pol II across this actively transcribed region^35^.

Ser-2 of the RNAPII Carboxy-Terminal Domain (CTD) becomes phosphorylated during initiation of transcription, an effect that promotes processive elongation^39^. As expected, ChIP-qPCR analysis of phosphorylated Ser-2 (pS2) CTD showed enrichment on the LTR upon stimulation of T-cell signaling in wild-type cells (Fig. 7e). However, accumulation of pS2 modified RNAPII was significantly reduced in the *TRIM24* KO cell line (Fig. 7e). Phosphorylation of CTD Ser-2 is produced by CDK9 of P-TEFb^40^, and consequently we also examined whether the *TRIM24* KO affected recruitment of CDK9 to the LTR. In agreement with observations that P-TEFb is sequestered by the 7SK snRNP complex in unstimulated T cells^41^, ChIP-qPCR using antibodies against CDK9 revealed low levels of association at the LTR in untreated cells, but became significantly enriched at the core promoter (RBE1) in wild-type cells treated with PMA and ionomycin (Fig. 7f). In contrast, we did not observe a significant increase of CDK9 occupancy in stimulated *TRIM24* KO cells (Fig. 7f). Importantly, we do not observe an effect of the *TRIM24* knockout on expression of CDK9 or cyclin T1 proteins (Supplementary Fig. 6). Consistent with the above results, and previous observations^42^, we find that treatment of cells with a CDK9 inhibitor prevented reactivation of latent HIV-1 in response to treatment with PMA (Supplementary Fig. 7). Collectively, these findings indicate that TRIM24 mediates activation of HIV-1 expression by promoting recruitment of CDK9 to the viral promoter and facilitating the transition of RNAPII into an actively elongating complex.

### Loss of TRIM24 does not cause substantial effects on LTR-associated histone modification

**Fig. 8:**
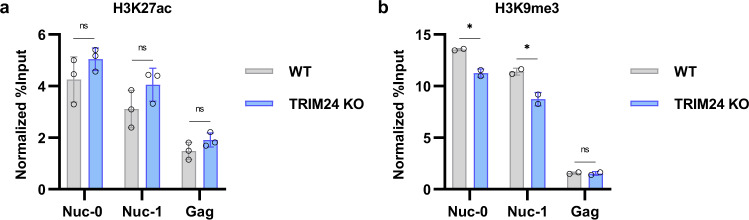
Loss of TRIM24 does not induce restrictive LTR chromatin organization. **a**, **b** Wild-type or *TRIM24* KO Jurkat mHIV-Luciferase cells were subject to ChIP-qPCR using antibodies targeting acetylated H3K27 (**a**) or trimethylated H3K9 (**b**). ChIP-qPCR results were normalized by subtraction of values produced with sample-paired non-specific IgG (*n* = 2–3, mean ± SD).

Upon integration into the host chromosome, the HIV-1 LTR becomes associated with two phased nucleosomes termed Nuc-0 and Nuc-1, positioned at −140 and the transcriptional start site^43,44^. These nucleosomes modified with H3K27ac is associated with activated transcription^45^, whereas the H3K9me3 modification is associated with transcriptional repression^28,46^. We examined the effect of *TRIM24* knockout on these modifications at the LTR and observed only a slight increase in H3K27ac (Fig. 8a) and a minor decrease in H3K9me3 upon *TRIM24* KO (Fig. 8b). These results indicate that loss of TRIM24 function does not cause significant alterations in chromatin modification at the LTR, and indicate its effect on HIV-1 transcription specifically involves a role in promoting transcriptional elongation.

### Regulation of HIV-1 by TRIM24 is independent of TRIM28

TRIM24 (TIF1α) along with the structurally similar TRIM28 (KAP1, TIF1β), TRIM33 (TIF1γ), and TRIM66 (TIF1δ) comprise the Transcription Intermediary Factor (TIF) subfamily of the Tripartite Motif (TRIM) family of proteins^20,47^. These proteins form regulatory complexes; in mice, TRIM24, TRIM28, and TRIM33 associate to suppress the development of hepatocellular carcinoma^22^, while human TRIM28 interacts with TRIM24 to promote prostate cancer tumorigenesis^48^. Of note, TRIM28 was implicated in the regulation of HIV-1 latency by modification of CDK9^49^, but overall the role of TRIM28 for regulation of HIV-1 transcription is contentious^36,37,50–52^. Because of these observations, we examined whether the association of TRIM28 with the HIV-1 LTR was dependent upon TRIM24. In this analysis we did not observe a difference in association of TRIM28 with the HIV-1 LTR in *TRIM24* KO cells compared to WT (Supplementary Fig. 8a). Furthermore, the *TRIM24* knockout did not alter the expression of *TRIM28* mRNA (Supplementary Fig. 8b) or protein (Supplementary Fig. 8c). These observations suggest that the effect of TRIM24 on the regulation of HIV-1 expression is independent of TRIM28.

### TFII-I and TRIM24 co-regulate specific subsets of cellular genes

Having discovered that TFII-I recruits TRIM24 to the HIV-1 LTR to promote transcriptional elongation, we next examined the extent that these factors contribute to co-regulation of cellular genes. Examination of publicly available ENCODE TFII-I and TRIM24 ChIP-seq datasets derived from the K562 lymphoblast cell line^53,54^ identified genomic binding sites of TRIM24 and TFII-I, for which the latter displayed notably higher coverage (Supplementary Fig. 9a, b). Comparison of the genomic loci of these factors revealed that just under 10% of TRIM24 bound sites were co-occupied with TFII-I (Supplementary Fig. 9a). Analysis of binding sites in relation to the nearest gene revealed a predilection for introns, intergenic, and promoter regions for both TFII-I and TRIM24 (Supplementary Fig. 9c), findings that are consistent with previous ChIP-seq experiments performed in non-K562 cell lines^48,55^. Strikingly, analysis of sites co-occupied by TFII-I and TRIM24 resulted in an enriched preference for promoters (Supplementary Fig. 9c), indicating that cellular gene regulation by TFII-I/TRIM24 potentially requires proximity to transcriptional start sites.

**Fig. 9:**
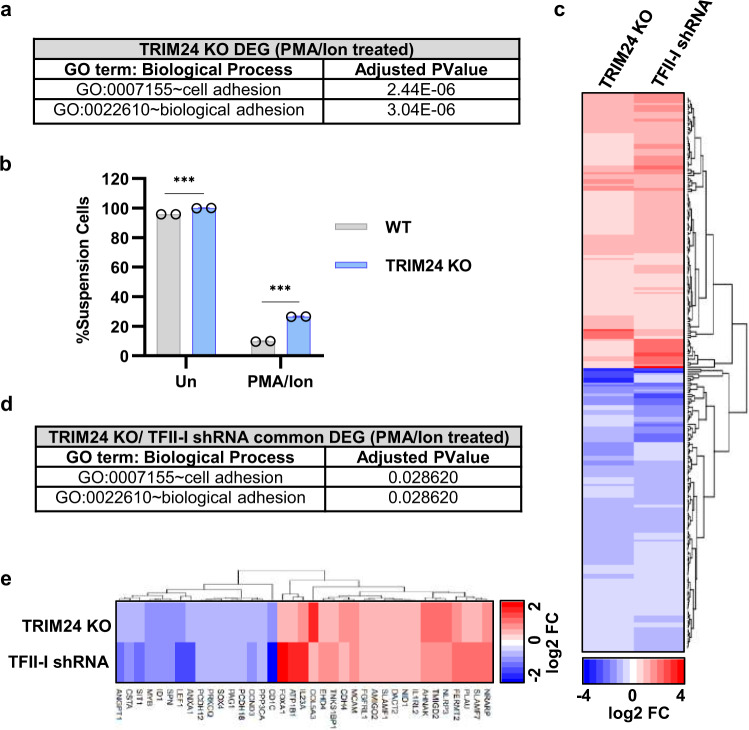
TFII-I enhances the TRIM24 transcriptional program. **a** PMA/ionomycin-treated *TRIM24* KO DEG display enrichment of cell adhesion-related biological processes as identified with DAVID online tool gene ontology analysis. **b** Wild-type or *TRIM24* KO Jurkat Tat T cells were left untreated or stimulated with 20 nM PMA/1 μM ionomycin for 2 h. The proportion of cells that adhered to the flask culture surface or remained in suspension was determined (*n* = 2, mean ± SD). **c** Heatmap depiction of significantly downregulated (blue) or upregulated (red) genes that are common to *TRIM24* KO and TFII-I knockdown cells (PMA/ionomycin-treated). Relative gene expression was determined by DESeq2 analysis of *n* = 3 RNA-seq samples. **d** DAVID online tool gene ontology analysis of *TRIM24* KO and TFII-I knockdown common DEG (PMA/ionomycin-treated) identified enrichment of cell adhesion-related biological processes. **e** Heatmap showing significantly downregulated (blue) or upregulated (red) genes that are common to TRIM24 KO and TFII-I knockdown (PMA/ionomycin-treated) and are involved in cellular adhesion. *n* = 3 RNA-seq samples were analyzed by DESeq2 to determine relative gene expression.

To further examine the role of TRIM24 and TFII-I for gene expression, we used RNA-Seq to identify differentially expressed genes (DEG) in PMA/ionomycin-activated Jurkat T cells depleted of *TRIM24* by gene knockout or TFII-I using shRNA. In this analysis, we identified 823 genes that were differentially expressed upon knockout of *TRIM24* (Supplementary Fig. 10a and Supplementary Data 2), and 2588 genes altered upon TFII-I knockdown (Supplementary Fig. 10b and Supplementary Data 2). In agreement with their function as activators or repressors, we observed near equal distribution between up- and downregulated genes upon factor depletion (Supplementary Fig. 10). Next, we examined the global function of TRIM24-responsive genes by performing Database for Annotation, Visualization, and Integrated Discovery (DAVID) analysis to determine enrichment of biological process gene ontology (GO) terms. Our RNA-seq analysis identified DEG between wild-type and *TRIM24* KO T cells treated with PMA and ionomycin, and consistently we observe enrichment of GO terms related to T-cell activation, including regulation of signal transduction, leukocyte activation and cytokine production (Supplementary Data 3), indicating a role for TRIM24 in T-cell immune response. We also observed GO terms related to cell adhesion (Fig. 9a and Supplementary Data 3), and although Jurkat T cells grow in suspension they adhere to culture surfaces upon activation of T-cell signaling^56,57^. Consistently, we observe that *TRIM24* KO Jurkat cells are significantly less adherent upon treatment with PMA and ionomycin compared to wild-type cells (Fig. 9b). It is well-established that expression of cell adhesion molecules are an important component of T-cell-mediated immune response^58–60^. In contrast, *TRIM24* KO does not abrogate global T-cell activation, as the majority of T-cell activation-induced and repressed genes are similarly regulated in *TRIM24* KO cells compared to wild-type (Supplementary Fig. 11c).

Because of the relationship between TFII-I and TRIM24 for the regulation of HIV-1, we examined the extent that these factors regulate common cellular transcriptional programs by analysis of genes whose differential expression were similar upon loss of TRIM24 or TFII-I. We found that a significant proportion of *TRIM24* KO DEG exhibited similar regulation patterns as did TFII-I knockdown, with 123 common downregulated genes (24.9% of *TRIM24* KO downregulated genes) and 119 common upregulated genes (36.1% of *TRIM24* KO upregulated genes) (Fig. 9c and Supplementary Fig. 12). DAVID analysis of these similarly regulated genes represented many of the same GO terms as *TRIM24* KO DEG (Supplementary Data 3), indicating that TFII-I largely facilitates the TRIM24 transcriptional program. Of note, genes that are dysregulated similarly with the loss of TRIM24 or TFII-I highlight cellular adhesion (Fig. 9d), as 37 of the common DEG are involved in adhesion with nearly half upregulated or downregulated (Fig. 9e). These 37 genes represent 31% of all genes involved in cell adhesion that are mis-regulated upon loss of TRIM24 (Supplementary Fig. 13 and Supplementary Data 4). Collectively, these results highlight that TFII-I augments the genome-wide TRIM24 transcriptional program, and indicates that regulation of many cellular genes may involve direct recruitment of TRIM24 by TFII-I.

## Discussion

In this study, we identified an interaction between TFII-I and TRIM24, which is required for efficient transcriptional elongation from the HIV-1 LTR promoter. The important role of transcriptional elongation for regulation of HIV-1 expression was recognized since the discovery that the viral transactivator Tat recruits P-TEFb to paused RNA Polymerase II complexes through interaction with nascent TAR RNA^40,61^. Accordingly, loss of Tat in cells bearing transcriptionally silenced provirus is thought to represent a significant barrier for reactivation of virus expression^62,63^. One report has indicated that several commonly studied latency-reversing agents (LRAs), including panobinostat and PEP005, cause reactivation of viral expression by promoting transcriptional elongation and splicing of sub-genomic transcripts, rather than initiation of transcription^34^, although mechanism(s) for this effect were not determined. Our results indicate that Tat or TAR are not required for stimulation of HIV-1 transcription by TRIM24. Consequently, it is possible that the effect of TFII-I-TRIM24 for recruitment of CDK9, and phosphorylation of CTD S2, might represent a priming mechanism to kick start synthesis of elongated and spliced transcripts for the production of Tat protein^64^ which would produce the characteristic positive feedback loop for viral gene expression^65–67^.

The function of TFII-I for the regulation of HIV-1 transcription has been enigmatic. Binding sites for TFII-I, in conjunction with USF1 and 2 are highly conserved on LTRs from patients^10^, and these elements are necessary for the reactivation of HIV-1 transcription in response to T-cell signaling^12^. This observation is consistent with results indicating that TFII-I is involved in activation of *c-Fos* expression in response to MAPK signaling^68^, but a specific mechanistic role of TFII-I for stimulation of transcription from the HIV-1 LTR has not been identified. TFII-I interacts with multiple additional DNA binding factors on various cellular promoters, including STAT1, STAT3, SRF, ERSF, and on the HIV-1 LTR with USF1 and USF2^12,16^. In the latter case, interaction of USF1/2 with the upstream RBE3 element on the HIV-1 LTR requires TFII-1 to produce a specificity unique to this complex^14^. These observations suggest that TFII-I plays a significant role for directing cooperative interaction of various sequence-specific factors with regulatory *cis*-elements. Our finding that TFII-I recruits the coactivator TRIM24 suggests this factor plays an additional more elaborate role for gene regulation than acting as a chaperone for DNA binding partners. It will be interesting to determine how additional functions attributed to TRIM24 are separated from those involved in interaction with TFII-I and recruitment to the HIV-1 LTR.

**Fig. 10:**
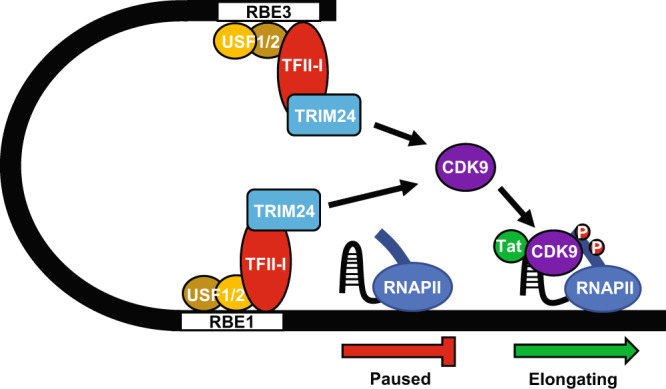
TRIM24 is an RBF-2 cofactor that stimulates transcriptional elongation of HIV-1. The TFII-I component of RBF-2 directly interacts with, and recruits TRIM24 to the HIV-1 LTR. TRIM24 is essential for T-cell signal-induced activation of the chromosomally integrated provirus and is required for recruitment of P-TEFb/CDK9 to the LTR, and phosphorylation of Ser-2 of the RNA Pol II CTD, to promote transcriptional elongation.

TRIM24 was previously identified as a co-activator for nuclear receptors, including for estrogen^69^, androgen^70^, and androstane^71^. However, the mechanism(s) by which TRIM24 causes transcriptional activation has not been identified. Considering our results, we propose that TRIM24 may also activate transcriptional elongation for nuclear receptor target genes. Our results indicate that TRIM24 enhances association of CDK9 to the HIV-1 LTR, and the simplest possibility is that TRIM24 may directly interact with CDK9 or cyclin T to promote the recruitment of P-TEFb (Fig. 10). Alternatively, TRIM24 may modify additional factors at the LTR core promoter to enable access or recruitment of P-TEFb. TRIM24 has E3-ubiquitin ligase activity and was shown to promote degradation of p53 through direct ubiquitylation^72,73^. TRIM24 was also found to modify the functions of CBP and TRAF3 by catalyzing K63-linked ubiquitination. TRIM24-mediated K63 ubiquitination of CBP inhibited macrophage polarization^74^, while this modification of TRAF3 was also observed as part of the anti-viral response to VSV infection^75^. Elucidation of the precise role of TRIM24 as a transcriptional co-activator will require a detailed understanding of its interactions with the general transcription factor machinery.

It is believed that TRIM24 contributes to cancer progression by operating as a coactivator for nuclear hormone receptors^21,23^. However, the majority of chromatin-associated TRIM24 is not co-localized with estrogen receptor (ER) in breast cancer tissue, and overexpression of TRIM24 correlates with poor patient survival independent of ER status^21^. Here, we found that TFII-I and TRIM24 display a substantial degree of genome-wide co-localization and that loss of TRIM24 alters the immune response, displayed in part by decreased cellular adhesion. A role for TRIM24 in immune function has previously been identified in that T-helper 2 cells deficient of TRIM24 are refractory to house dust mite-induced allergic inflammatory activation, although a mechanism for this effect was not determined^76^. Furthermore, MCF7 breast cancer cells deficient in TRIM24 also display a reduced cell adhesion phenotype that is thought to contribute to cancer cell proliferation, migration, and metastasis^27^. Consequently, we believe it unlikely that TRIM24-regulated transcriptional programs such as cancer and inflammation are the sole result of nuclear receptor co-activator function, and that gene regulation by TRIM24 occurs to some degree through direct recruitment by additional DNA binding factors, including TFII-I.

There is currently considerable interest in the development of therapies that could be applied in addition to, or instead of, ART to eliminate latently infected cells from patients. One potential strategy, broadly termed “block and lock” is based on the rationale that preventing stochastic basal transcriptional noise of the latent provirus may discourage maintenance of the latently infected cell population^4,77^. Based on the results presented here, we suggest that TRIM24, and specifically the interaction between TRIM24 and TFII-I may represent an important specific target for this strategy.

## Methods

### Cell and virus culture

Jurkat E6-1, Jurkat Tat, and TZM-bl cells were cultured under standard conditions as previously described^17^. Vesicular stomatitis virus G (VSV-G) pseudotyped viral stocks were produced by co-transfecting HEK293T cells with a combination of viral molecular clone, psPAX, and pHEF-VSVg as previously described^17^.

### shRNA knockdown

Jurkat mHIV-Luciferase or JLat10.6 cells were infected with pLKO empty vector or pLKO shRNA expressing lentivirus at a M.O.I. ~10. For Jurkat mHIV-Luciferase cells, TFII-I shRNA-infected cells were cultured for 8 days with 7.5 µg/mL puromycin, while TRIM24 shRNA-infected cells were cultured for 3 days with 3 µg/mL puromycin. For JLat10.6 cells, all shRNA-infected cells were cultured 4 days with 1 µg/mL puromycin. Pools of puromycin-selected cells were prepared for the indicated analysis. MISSION shRNA clones (Sigma) used for knockdown in Jurkat mHIV-Luciferase cells were as follows: TFII-I, TRCN0000019315; TRIM24-1, TRCN0000021263; TRIM24-2, TRCN0000021262; TRIM24-3, TRCN0000021259. MISSION shRNA clones (Sigma) that were used for knockdown in JLat10.6 cells were: TFII-I, TRCN0000364550; TRIM24, TRCN0000021262.

### TRIM24 knockout

*TRIM24* KO cell lines were generated using CRISPR-Cas9 in the Jurkat Tat mHIV-Luciferase cell line that possesses a chromosomally integrated HIV-1 mini-virus where luciferase is expressed from the 5’ LTR (Fig. 1a). Briefly, 2 × 10^6^ cells were co-transfected with Cas9 (pU6_CBh-Cas9-T2A-BFP: Addgene #64323) and gRNA (pSPgRNA: Addgene #47108) sequences that target genomic *TRIM24*, using the Neon Transfection System (Invitrogen) as per the manufacturer’s instructions with the following settings: voltage, 1350 V; width, 20 ms; pulse number, 3×. Knockout cells were isolated by live sorting (Astrios Flow Cytometer) BFP positive cells into 96-well plates containing complete RPMI 1640. Clones were expanded, and knockout of *TRIM24* was validated by PCR genotyping and western blotting. *TRIM24* gRNA target sequences were CTGCATATTATTTAAGCAAC and GAACGAGGCCGAGAGTCGGC.

### Immunoblotting and immunoprecipitation

Western blotting was performed as previously described^78^. Antibodies were as follows: Tubulin (1:40,000)—Abcam ab7291; TFII-I (1:20,000)—Abcam ab134133; Flag (1:20,000)—Sigma-Aldrich F3165; Myc (1:2500)—Santa Cruz sc-40; TRIM24 (1:4000)—Proteintech 14208-1-AP; KAP1 (1:2500)—Proteintech 15202-1-AP; GAPDH (1:10,000)—Abcam ab9484; CDK9 (1:4000)—Abcam ab239364; Cyclin T1 (1:500)—Santa Cruz sc-10750; streptavidin-HRP (1:40,000)—Abcam ab7403; goat anti-rabbit-HRP (1:2,000,000)—Abcam ab6721; goat anti-mouse-HRP (1:20,000)—Pierce #1858413.

Immunoprecipitations (IPs) were performed using HEK293T or Jurkat E6-1 cells. In total, 8.33 × 10^5^ HEK293T cells were plated with 2 mL DMEM in six-well plates. The following day, cells were transfected with 4 μg of the indicated construct, using 3 µg PEI per 1 µg plasmid DNA^79^, and harvested 24–48 h post transfection. For Jurkat IPs, cells were transduced with TRIM24-Flag or empty vector lentivirus and experiments were performed following puromycin selection. Cells were collected, washed with ice-cold PBS, and suspended in DR Buffer A (10 mM HEPES-KOH pH  = 7.9, 10 mM KCl, 1.5 mM MgCl_2_, 0.5 mM DTT, 1× PIC, 0.5 mM PMSF) and incubated at 4 °C for 15 min. Samples were centrifuged, the cytoplasmic supernatant was discarded while the pelleted nuclei were suspended in DR Buffer C (20 mM HEPES-KOH pH = 7.9, 0.42 M NaCl, 1.5 mM MgCl_2_, 0.2 mM EDTA, 25% glycerol, 0.5 mM DTT, 1× PIC, 0.5 mM PMSF) and briefly sonicated (Covaris S220 Focused-ultrasonicator). Samples were cleared by centrifugation and protein concentrations were determined using a Bradford assay (BioRad). In all, 250 μg of nuclear extract was diluted with Flag-IP Buffer (50 mM Tris-HCl pH = 8.0, 90 mM NaCl, 1 mM EDTA, 1% Triton X-100, 1× PIC) and 2 μg of antibody was added (Flag—Sigma-Aldrich F3165; mouse IgG—Santa Cruz sc-2025); the antibody–lysate mixture was incubated at 4 °C with rotation for 1 h prior to the addition of protein A/G agarose beads (Millipore, 50 μL/IP) and continued overnight incubation. The samples were washed 3× with Flag-IP Buffer and eluted in 4× SDS Sample Buffer with boiling. Eluted proteins were subject to analysis by immunoblotting.

### Chromatin immunoprecipitation

Exponentially growing Jurkat mHIV-Luciferase or mdHIV Clone 11 cells (3 × 10^7^ cells/IP) were fixed with 1% formaldehyde (Sigma-Aldrich) for 10 min at room temperature. Cross-linking was quenched with 125 mM glycine for 5 min, at which point cells were collected and washed with ice-cold PBS. Cells were incubated in NP-40 Lysis Buffer (0.5% NP-40, 10 mM Tris-HCl pH = 7.8, 3 mM MgCl_2_, 1× PIC, 2.5 mM PMSF) for 15 min on ice. Following sedimentation, the supernatant was discarded, and the pellet was resuspended in Sonication Buffer (10 mM Tris-HCl pH  = 7.8, 10 mM EDTA, 0.5% SDS, 1× PIC, 2.5 mM PMSF). Nuclei were sonicated using a Covaris S220 Focused-ultrasonicator to produce sheared DNA between 2000 and 200 bp. Samples were pelleted, with the soluble supernatant collected as the chromatin fraction and snap-frozen in liquid nitrogen. Chromatin concentrations were normalized among samples and pre-cleared with Protein A/G agarose (Millipore, 100 μL/IP). The chromatin samples were split in two and diluted with IP buffer (10 mM Tris-HCl pH = 8.0, 1.0% Triton X-100, 0.1% deoxycholate, 0.1% SDS, 90 mM NaCl, 2 mM EDTA, 1× PIC). The indicated specific antibody was added to one sample while control IgG was added to the other and the chromatin/antibody mixture was incubated 1 h at 4 °C with rotation. Antibodies used are as follows: TFII-I (10 μg)—BD Biosciences 610942, TRIM24 (10 μg)—Proteintech 14208-1-AP, RNAPII (5 μg)—Abcam ab26721, RNAPII pS2 (5 μg)—Abcam ab238146, CDK9 (10 μg)—Abcam ab239364, NFκB p65 (5 μg)—Thermo Fisher 51-0500, KAP1 (10 μg)—Abcam ab10483, H3K27ac (5 μg)—Abcam ab4729, H3K9me3 (5 μg)—Abcam ab176916, Mouse IgG (equivalent μg)—Santa Cruz sc-2025, Rabbit IgG (equivalent μg)—Abcam ab1722730. Pre-washed Protein A/G agarose beads (40 μL/IP) were then added to the samples and incubated overnight at 4 °C with rotation. Bead–antibody complexes were washed 3× in Low Salt Wash Buffer (20 mM Tris-HCl pH = 8.0, 0.1% SDS, 1.0% Triton X-100, 2 mM EDTA, 150 mM NaCl, 1× PIC) and 1× with High Salt Wash Buffer (same but with 500 mM NaCl). Elution and crosslink reversal was performed by incubating 4 h at 65 °C in EB supplemented with RNase A. DNA was purified using the QIAQuick PCR purification kit (QIAGEN) and ChIP DNA was analyzed using the Quant Studio 3 Real-Time PCR system (Applied Biosystems). Oligos used for ChIP-qPCR are as follows; RBE3/Nuc-0: fwd 5’-AGCCGCCTAGCATTTCATC-3’, rev 5’-CAGCGGAAAGTCCCTTGTAG-3’. RBE1/Nuc-1: fwd 5’-AGTGGCGAGCCCTCAGAT-3’, rev 5’-AGAGCTCCCAGGCTCAAATC-3’. Gag: fwd 5’-AGCAGCCATGCAAATGTTA-3’, rev 5’-AGAGAACCAAGGGGAAGTGA-3’. NFκB Enhancer Region (NER): fwd 5’-TTTCCGCTGGGGACTTTC-3’, rev 5’ CCAGTACAGGCAAAAAGCAG-3’.

### Bio-ID assays

In all, 8.33 × 10^6^ HEK293T cells were plated in 2 mL DMEM in six-well plates and incubated overnight. Cells were transfected using PEI with 3 μg of the indicated TurboID construct and incubated overnight. Biotin was added directly to the media at a final concentration of 500 μM, and cells were incubated for 1 h. Biotinylation was stopped by washing cells 2× in ice-cold PBS. Endogenous TRIM24 was immunoprecipitated as described above (2 μg of TRIM24—Proteintech #14208-1-AP), and samples analyzed by immunoblotting.

### Luciferase reporter assays

Transient luciferase expression assays in HEK293T cells were performed as previously described^17^. Briefly, transfections were performed in 96-well plates seeded with 2 × 10^4^ HEK293T cells per well 24 h prior to transfection. In total, 10 ng of pGL3 reporter plasmid along with 10 ng of either pcDNA3.1 + (Invitrogen) or pcDNA-Tat and 100 ng expression vector was co-transfected. Luciferase activity was measured 24 h post transfection. For Jurkat luciferase reporter assays, 1 × 10^5^ luciferase expressing Jurkat cells were plated with 100 µL media in 96-well plates. Luciferase activity was measured after the indicated time of treatment. Measurements were performed using Superlight^TM^ luciferase reporter Gene Assay Kit (BioAssay Systems) as per the manufacturer’s instructions; 96-well plates were read in a VictorTM X3 Multilabel Plate Reader.

### Q-RT-PCR

RNA was extracted from Jurkat mHIV-Luciferase cells following the indicated treatment using RNeasy Kit (Qiagen). RNA was analyzed using the Quant Studio 3 Real-Time PCR system (Applied Biosystems) using *Power* SYBR® Green RNA-to-CT™ 1-Step Kit (Thermo Fisher) as per the manufacturer’s instructions. Primers for analysis of HIV transcripts were as follows: Initiation, fwd 5’-GTTAGACCAGATCTGAGCCT-3’, rev 5’-GTGGGTTCCCTAGTTAGCCA-3’; Proximal, fwd 5’-TGGGAGCTCTCTGGCTAACT-3’, rev 5’-TGCTAGAGATTTTCCACACTGA-3’; Gag, fwd 5’-CTAGAACGATTCGCAGTTAATCCT-3’, rev 5’-CTATCCTTTGATGCACACAATAGAG-3’.

### ChIP-seq analysis

Call sets were downloaded from the ENCODE website (https://www.encodeproject.org/). Library identifiers are as follows; ENCLB915UWS, ENCLB381ZDN, ENCLB949IYC, ENCLB887DPL. Heatmaps were generated from the ENCODE data using the plotHeatmap program.

### RNA-seq

RNA was extracted from wild-type, *TRIM24* KO, LKO control shRNA transduced, or TFII-I shRNA transduced Jurkat T cells that were untreated or treated with 20 nM PMA/1 μM ionomycin for 4 h. Sample quality control was performed using a Agilent 2100 Bioanalyzer. Qualifying samples were then prepped following the standard protocol for the NEBnext Ultra ii Stranded mRNA (New England Biolabs). Sequencing was performed on the Illumina NextSeq 500 with Paired-End 42 bp × 42 bp reads. De-multiplexed read sequences were uploaded to the Galaxy web platform^80^ and aligned to the hg38 reference genome using STAR; featureCounts determined transcript assembly, and differential gene expression (DEG) was determined by DESeq2 analysis. *TRIM24* KO and TFII-I knockdown DEG are defined as having a fold change in expression >1.5 and *P* value <0.05. The criteria for genes identified as responsive to T-cell activation are fold change >2, *P* value <0.05. Heatmaps were created in RStudio.

### Cell adherence assay

Overall, 2 × 10^6^ wild-type or TRIM24 KO Jurkat mHIV-Luciferase cells were plated in six-well plates with 2 mL RPMI. Cells were left untreated or treated with 20 nM PMA and 1 µM ionomycin and incubated for 2 h. The overlayed media was collected and the cells within were counted as the proportion of cells suspended. The cells that remained on the culture plates were scraped, resuspended in media, and counted as the adherent population, using a BioRad TC20 Automated Cell Counter.

### Flow cytometry

Cells were treated as indicated in the figure legends. Following the indicated treatment, cells were suspended in PBS and analyzed on a BD Biosciences LSRII-561 system where threshold forward scatter (FSC) and side scatter (SSC) parameters were set so that a homogenous population of live cells was counted (Supplementary Fig. 14). FlowJo software (TreeStar) was used to analyze data Data and determine to mean fluorescent intensity (MFI).

### Statistics and reproducibility

All replicates are independent biological replicates and are presented as mean values with ±standard deviation shown by error bars. The number of times that an experiment was performed is indicated in the figure legends. The two exceptions to this are Fig. 3f, WT–EV and Fig. 7b, TRIM24 KO–Gag, both of which had a single outlier value removed. *P* values were determined by performing unpaired samples *t* tests with the use of GraphPad Prism 9.0.0. Statistical significance is indicated at *P < 0.05, **P < 0.01, or ***P < 0.001, with n.s. denoting non-significant *P* ≥ 0.05.

### Reporting summary

Further information on research design is available in the Nature Portfolio Reporting Summary linked to this article.

## Supplementary information

Supplementary Information

Description of Additional Supplementary Data

Supplementary Data 1

Supplementary Data 2

Supplementary Data 3

Supplementary Data 4

Reporting Summary

## Data Availability

All data supporting the findings of this study are available within the article or from the corresponding author upon reasonable request. All high-throughput RNA-seq data (Supplementary Data 1) have been deposited to NCBI GEO and are publicly available under the accession codes GSE221633 and GSE221851.
