# Supplementary material for: TRIM24 controls induction of latent HIV-1 by stimulating transcriptional elongation
Source: Commun Biol. 2023 Jan 23;6:86. doi: 10.1038/s42003-023-04484-z (PMC9870992; doi:10.1038/s42003-023-04484-z)
Supplement: Supplementary file 1 — Supplementary Information [file 42003_2023_4484_MOESM1_ESM.pdf]

Supplementary Figures for "TRIM24 controls induction of latent HIV-1 by stimulating transcriptional elongation", Riley M. Horvath, Matthew Dahabieh, Tom Malcolm, and Ivan Sadowski

Supplementary Figure 1

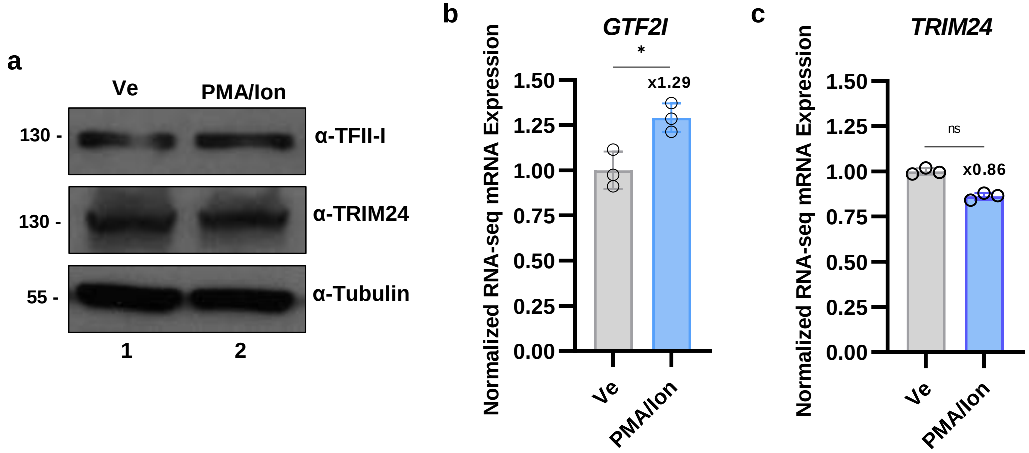

Supplementary Figure 1. Effect of T cell activation on TFII-I and TRIM24 expression. a: Following 4 hr treatment of Jurkat mHIV-Luciferase cells with a vehicle control (Ve, DMSO) or 20 nM PMA/ 1 μM ionomycin, whole cell lysates were analyzed by immunoblotting with antibodies targeting TFII-I, TRIM24, or Tubulin. **b, c:** Jurkat mHIV-Luciferase cells were incubated with a vehicle (Ve, DMSO) or 20 nM PMA/ 1 μM ionomycin for 4 hrs. RNA was extracted and RNA-seq was performed (*n* = 3). Normalized counts of TFII-I (*GTF2I*, **b**) and *TRIM24* mRNA (**c**) were produced by DESeq2 analysis.

Supplementary Figure 2

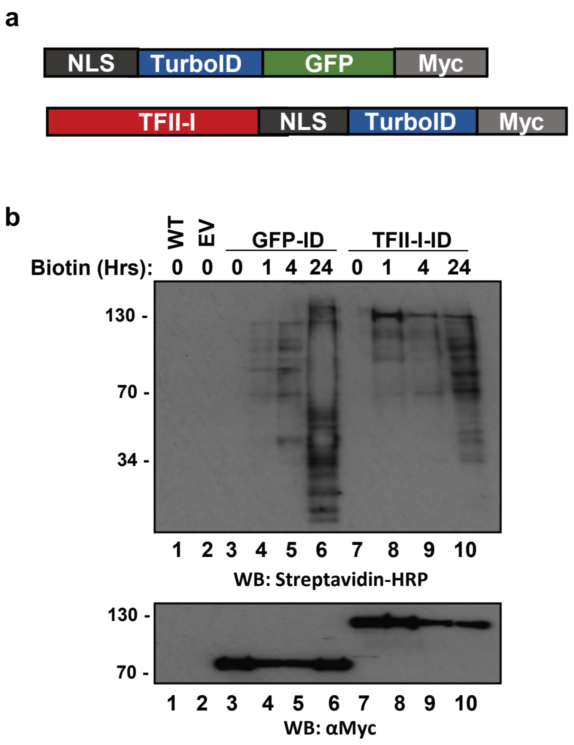

Supplementary Figure 2. TurboID fusion proteins expressed in HEK293 cells. a: Schematic representation of Myc epitope tagged GFP and TFII-I TurboID fusions. **b:** HEK293T cells (lane 1) were transfected with plasmids expressing GFP-TurboID (lanes 3-6), TFII-I-TurboID (lanes 7-10), or an empty vector control (EV, lane 2). Cells were incubated with 500 μM biotin for the indicated amount of time (lanes 3-10), when lysates were prepared and immunoblotted using Streptavidin-HRP or anti-Myc antibodies.

Supplementary Figure 3

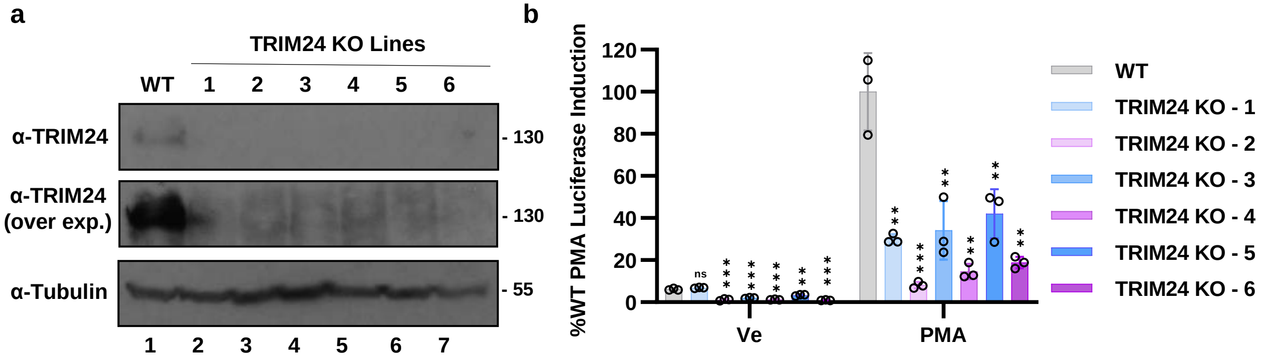

Supplementary Figure 3. *TRIM24* knockout inhibits HIV-1 expression. a: Knockout lines of *TRIM24* (lanes 2-7) were produced from Jurkat mHIV-Luciferase cells (lane 1) using CRISPR/Cas9. Following expansion of clones, lysates were analyzed by immunoblotting using antibodies against TRIM24 or Tubulin. **b:** Jurkat mHIV-Luciferase parent (WT) and *TRIM24* KO clonal cell lines were treated with a vehicle control (Ve, DMSO) or with 20 nM PMA for 4 hr, at which point luciferase activity was measured (n = 3, mean ± SD).

Supplementary Figure 4

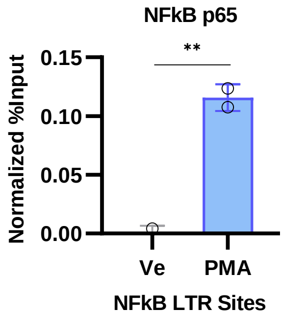

Supplementary Figure 4. NFκB p65 is recruited to the LTR upon T cell activation. ChIP qPCR analysis with antibodies against NFκB p65 was performed with Jurkat Tat mdHIV Clone 11 following vehicle control treatment (Ve, DMSO) or stimulation with 50 nM PMA for 24 hrs. Results were normalized by subtraction of values produced with sample paired non-specific IgG (n = 2, mean ± SD).

Supplementary Figure 5

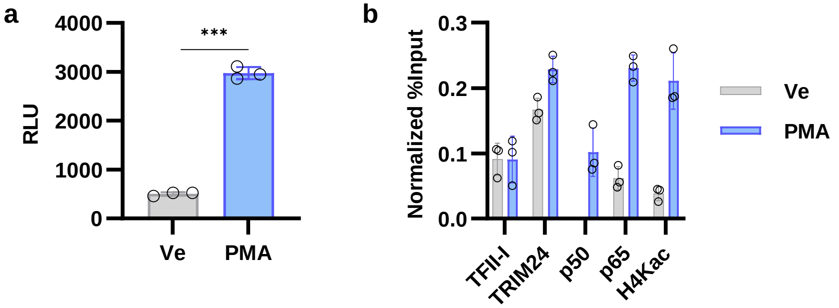

Supplementary Figure 5. TRIM24 and TFII-I co-localize to the LTR in HeLa cells. a: TZM-bl cells were treated with DMSO or PMA (100 ng/mL) for 4 hr prior to measurement of luciferase activity (*n* = 3, mean ± SD). **b:** ChIP-qPCR with TZM-bl cells treated with DMSO (Ve) or PMA was performed using the indicated antibodies. *n* = 3, mean ± SD, with normalization performed by subtracting IgG values.

Supplementary Figure 6

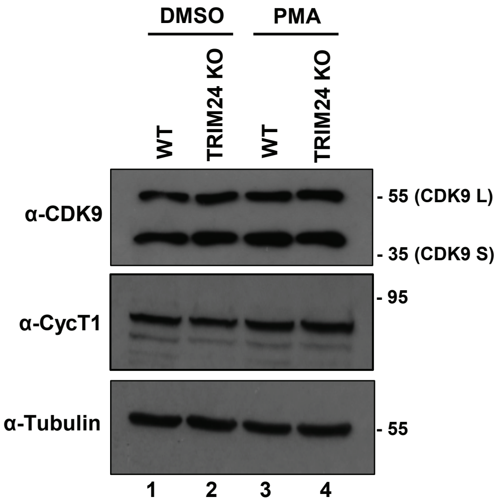

Supplementary Figure 6. TRIM24 depletion does not alter CDK9 or cyclin T protein levels. Wildtype or *TRIM24* KO Jurkat mHIV-Luciferase cells were left untreated (DMSO) (lanes 1-2) or treated with 20 nM PMA (lanes 3-4) for 4 hrs. Lysates were analyzed by immunoblotting with antibodies against CDK9, Cyclin T1, or Tubulin as indicated.

Supplementary Figure 7

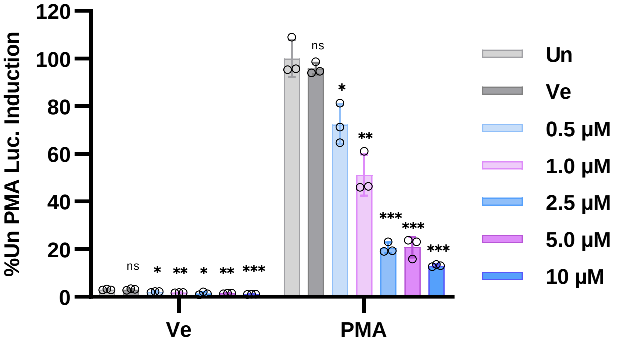

Supplementary Figure 7. CDK9 kinase activity is necessary for induction of HIV-1 expression. Following 1 hr pre-treatment with the indicated concentration of CDK9 kinase inhibitor LDC67, Jurkat mHIV-Luciferase cells were left untreated (DMSO) or incubated with 20 nM PMA for 4 hr prior to luciferase measurement (n = 3, mean ± SD).

Supplementary Figure 8

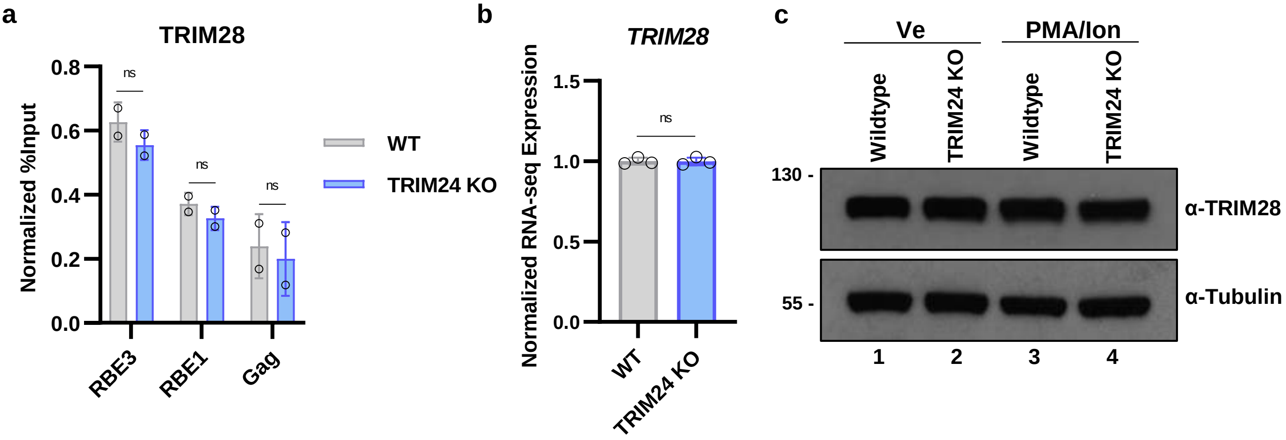

Supplementary Figure 8. TRIM24 regulates HIV-1 latency independently of TRIM28. a. ChIP-qPCR was performed on wildtype or *TRIM24* KO Jurkat mHIV-Luciferase cells using anti-TRIM28 antibody. Paired non-specific IgG values have been subtracted, $n = 2$, mean ± SD. **b:** RNA-seq was performed on mRNA extracted from wildtype or *TRIM24* KO Jurkat mHIV-Luciferase cells. Shown are the *TRIM28* mRNA normalized counts produced by DESeq2 analysis of 3 independent replicates ($n = 3$). **c:** Wildtype or *TRIM24* KO Jurkat mHIV-Luciferase cells were incubated with a vehicle control (Ve, DMSO) or 20 nM PMA/ 1 μM ionomycin for 4 hrs. Subsequently, whole cell lysates were extracted and subject to immunoblotting with antibodies against TRIM28 or Tubulin.

Supplementary Figure 9

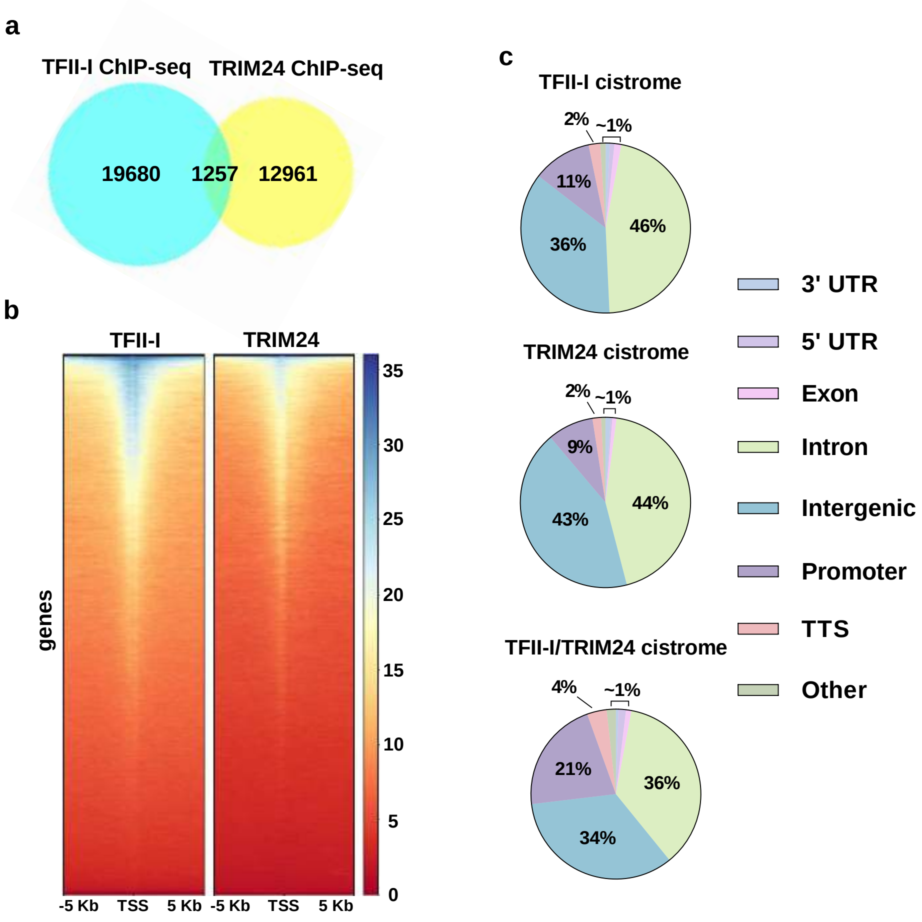

Supplementary Figure 9. Co-localization of TRIM24 and TFII-I in K562 cells. a: Venn diagram indicating overlap of ChIP-seq peaks derived from IP of eGFP-TFII-I or TRIM24 performed in K562 cells. **b:** Heatmap depiction of eGFP-TFII-I or TRIM24 binding sites obtained from ChIP-seq using the K562 cell line. **c:** Annotation of the genomic distribution of eGFP-TFII-I, TRIM24, and TFII-I/TRIM24 overlapping ChIP-seq peaks.

Supplementary Figure 10

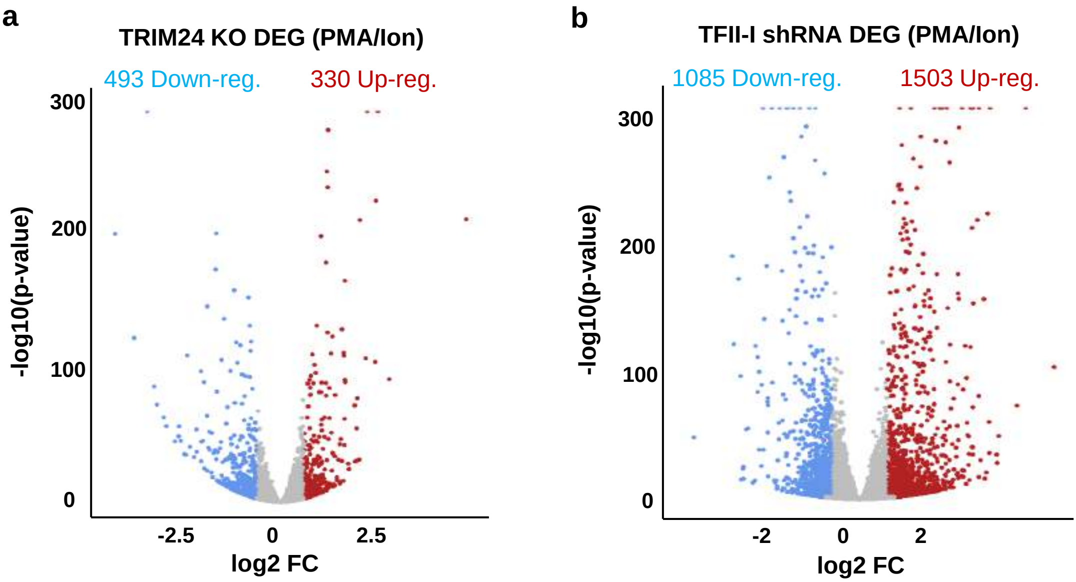

Supplementary Figure 10. TRIM24 KO and TFII-I knockdown differentially expressed genes (DEG). a, b: Volcano plot depicting DESeq2 analysis comparing wildtype to *TRIM24* KO (**a**) or control shRNA transduced cells to TFII-I shRNA transduced Jurkat mHIV-Luciferase cells (**b**). Samples were treated with 20 nM PMA/ 1 μM ionomycin for 4 hrs prior to RNA extraction. Analysis was performed on $n = 3$ RNA-seq samples with significant genes having p-value < 0.05 and fold change > 1.5.

Supplementary Figure 11

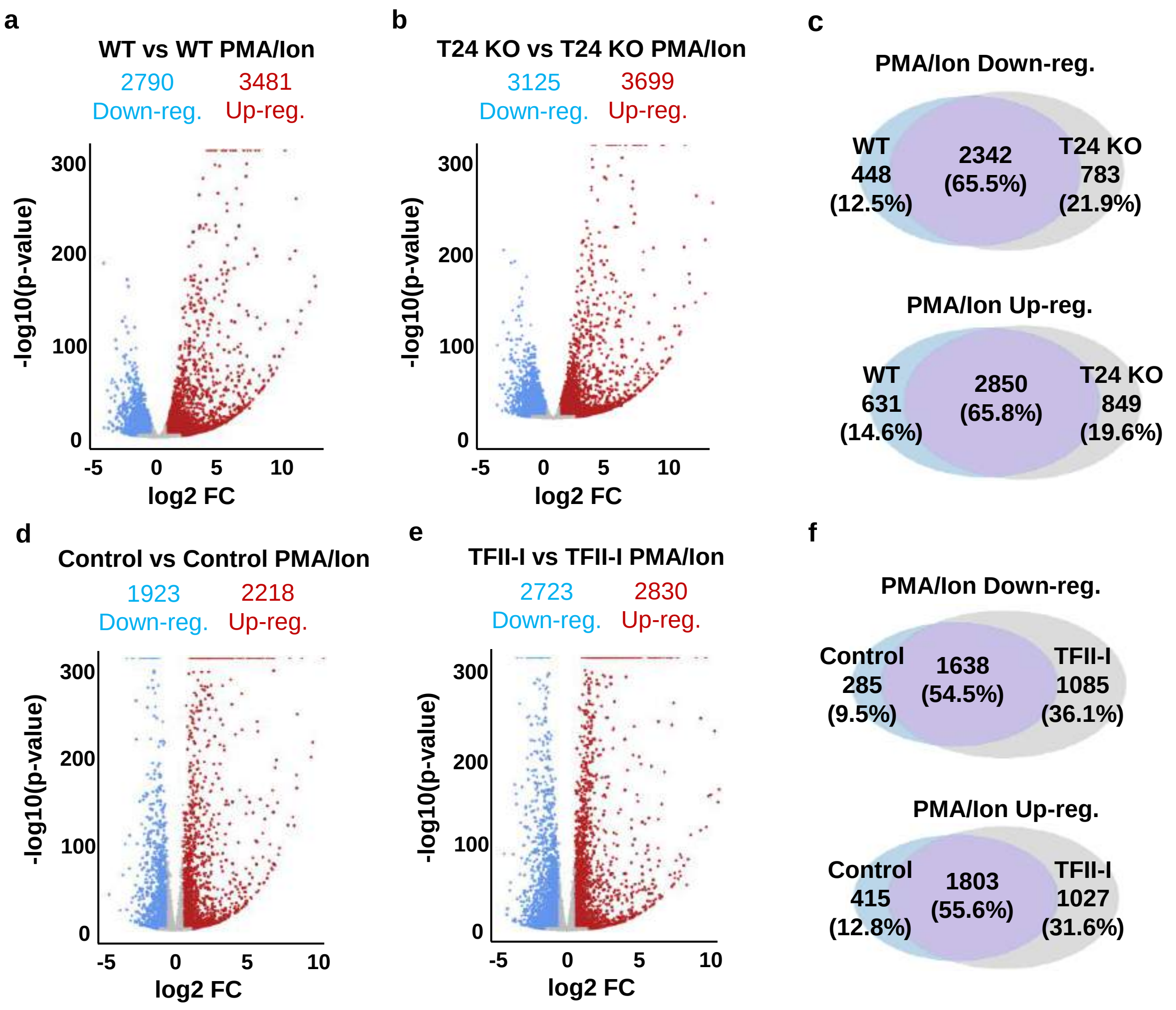

Supplementary Figure 11. Loss of TRIM24 or TFII-I does not prevent global T cell activation. a, b, d, e: Volcano plot depiction of DEG resultant from 4 hr treatment with 20 nM PMA/ 1 μM ionomycin of the indicated Jurkat mHIV-Luciferase cell lines. Genes that exhibited greater than 1.5-fold change and p-value < 0.05 upon PMA/ ionomycin treatment are defined as significant. DESeq2 analysis was performed on *n* = 3 RNA-seq samples. **c, f:** Venn diagram display of genes significantly repressed or activated by PMA and ionomycin treatment for the indicated cell line.

Supplementary Figure 12

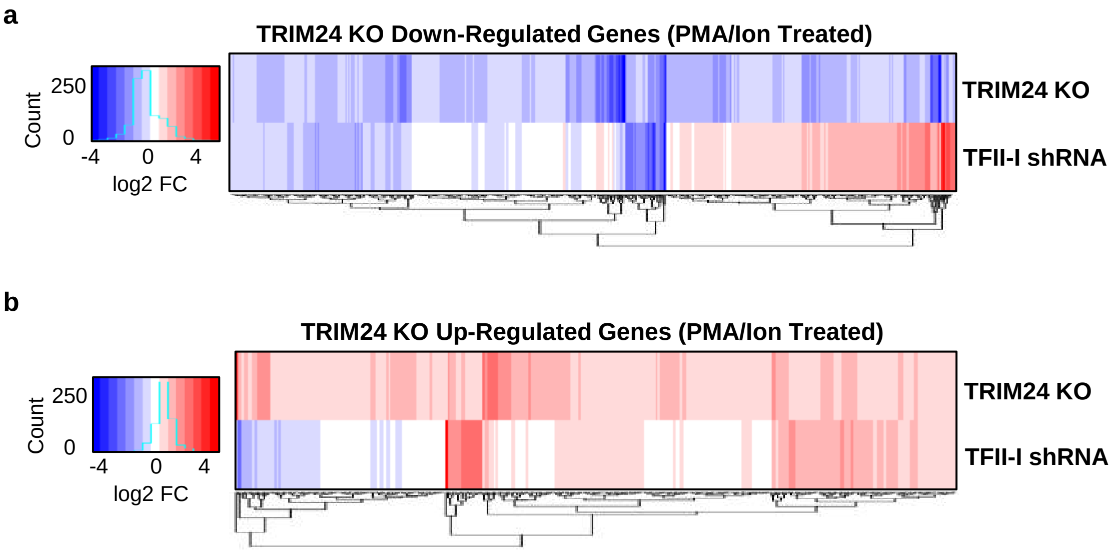

Supplementary Figure 12. *TRIM24* KO gene regulation is reflected by TFII-I knockdown. **a, b:** Heat map depictions of *TRIM24* KO down-regulated (**a**) and up-regulated (**b**) genes. Relative gene expression was determined by DESeq2 analysis of $n = 3$, mean ± SD RNA-seq samples that were treated with 20 nM PMA/ 1 μM ionomycin for 4 hrs prior to RNA extraction.

Supplementary Figure 13

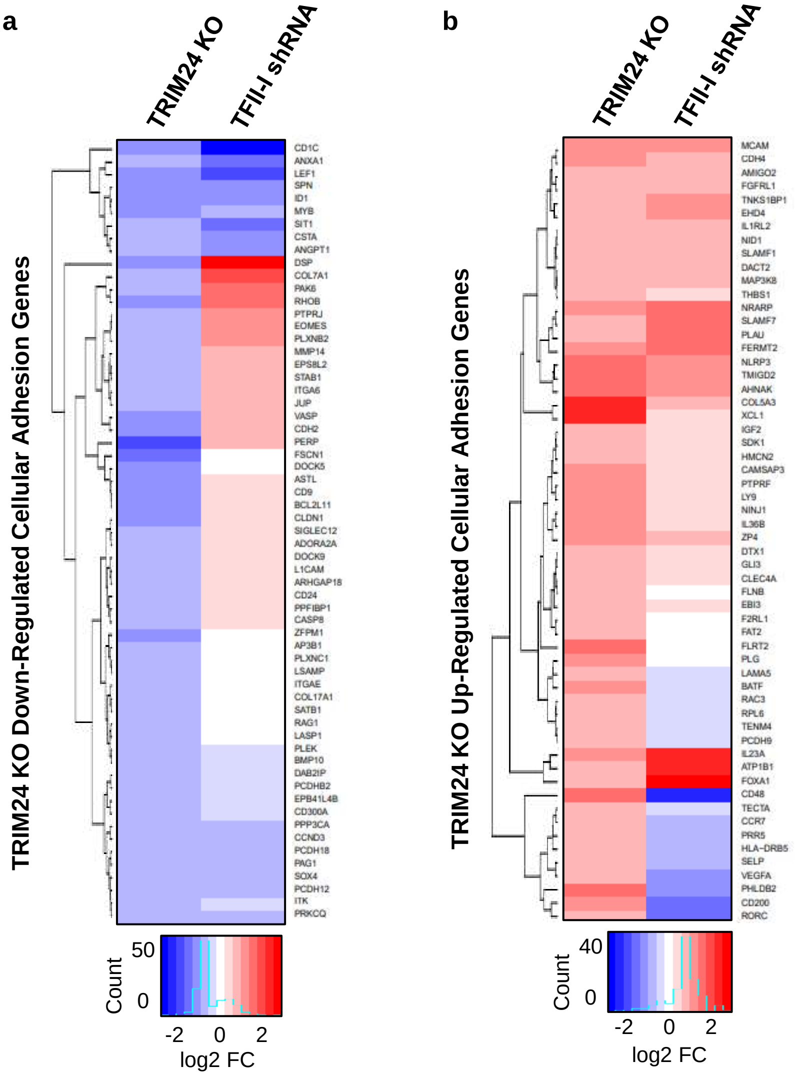

Supplementary Figure 13. TRIM24 regulated cellular adhesion genes are similarly affected by TFII-I knockdown. a, b: Heat maps depicting the expression of TRIM24 KO down-regulated (**a**) and up-regulated (**b**) cellular adhesion genes. Relative gene expression was determined by DESeq2 analysis of n = 3 RNA-seq samples that were treated with 20 nM PMA/ 1 μM ionomycin for 4 hrs prior to RNA extraction.

Supplementary Figure 14

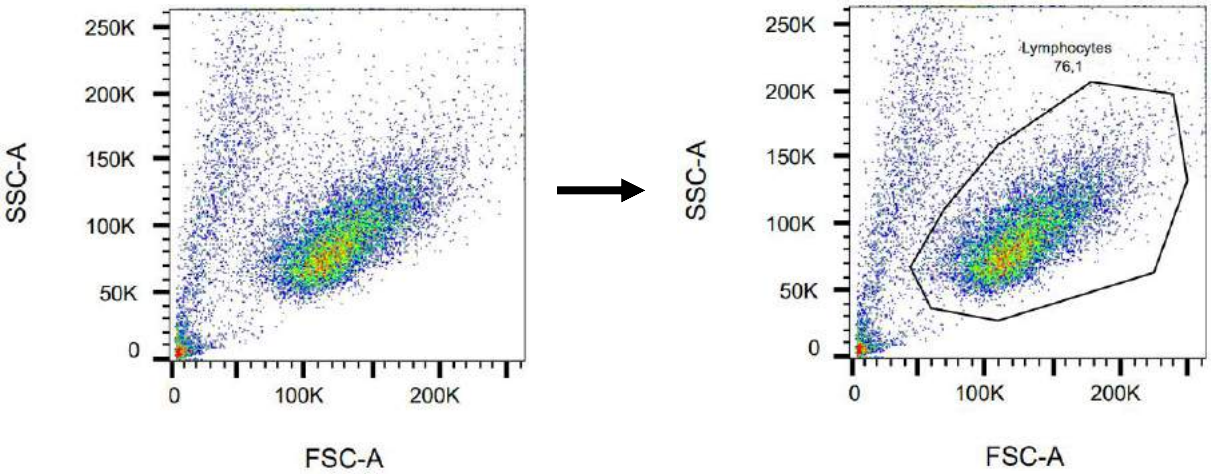

Supplementary Figure 14. Representative flow cytometry gating strategy. Representative scatter plots depicting the gating strategy employed. Homogeneous populations of live cells were isolated and assessed by setting threshold forward scatter (FSC) and side scatter (SSC) settings.

Supplementary Figure 15. Full length western blots.

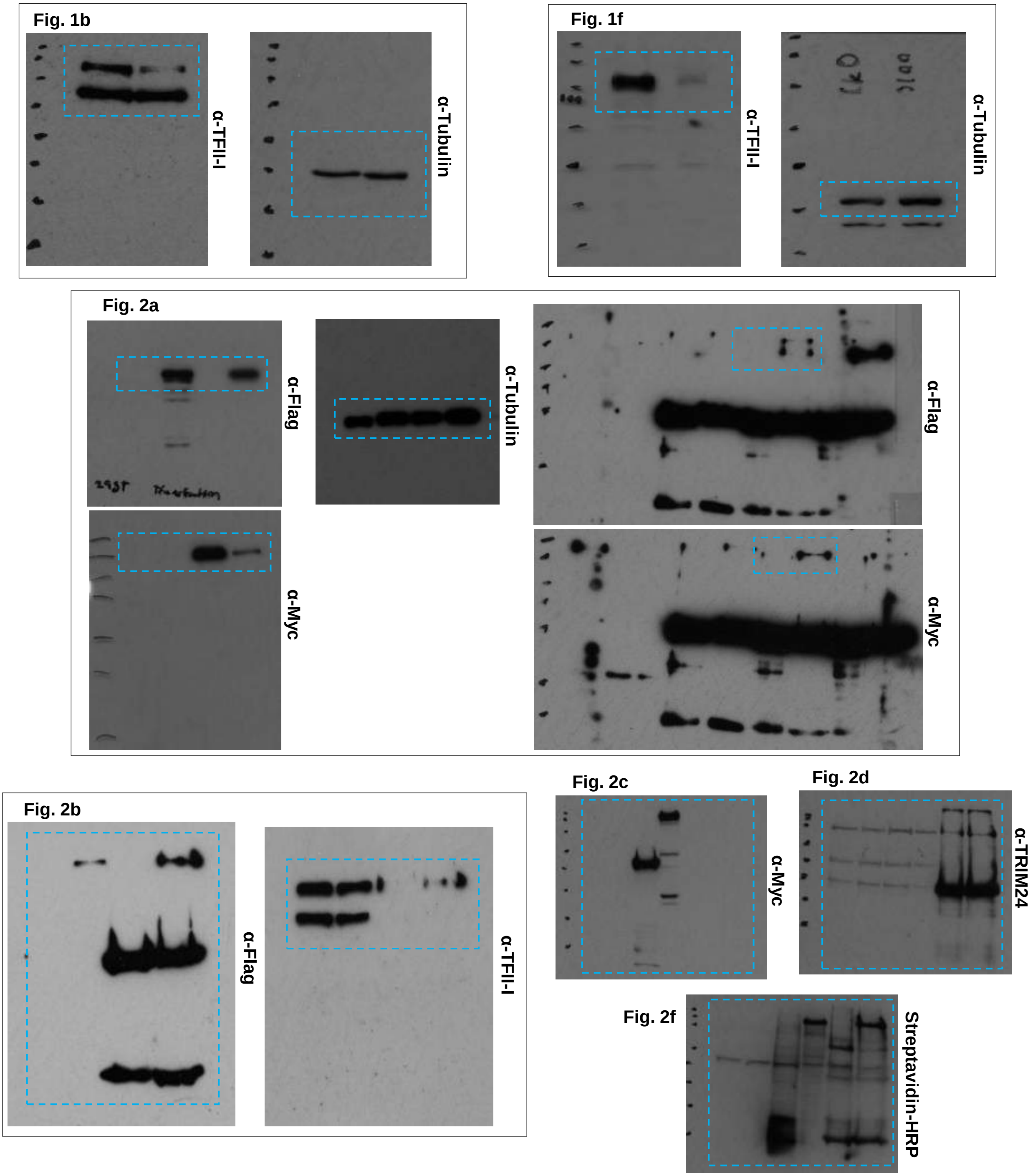

Fig. 3a

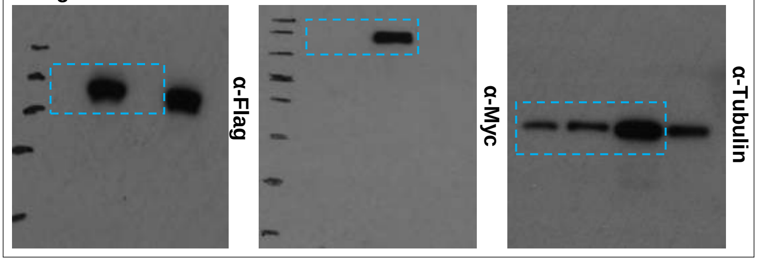

Fig. 3c

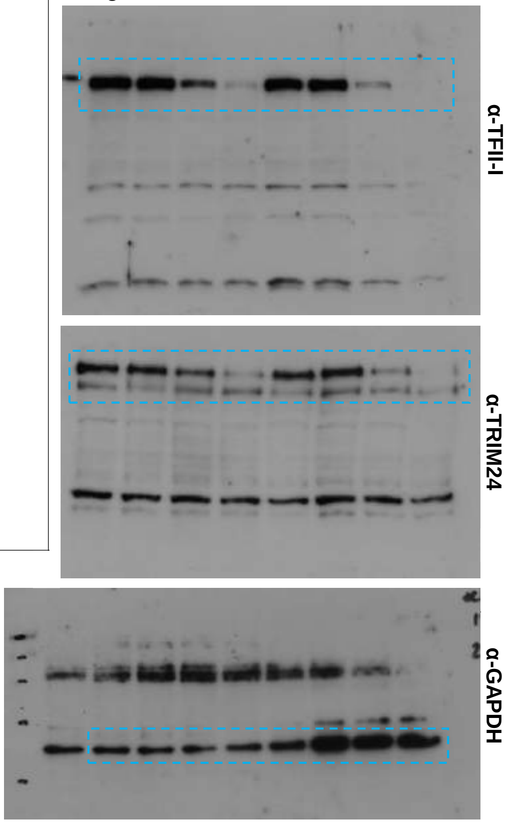

Fig. 4a

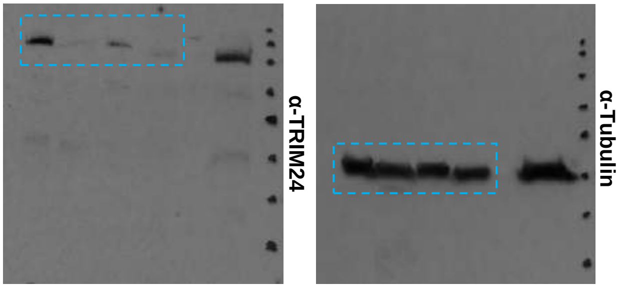

Fig. 4c

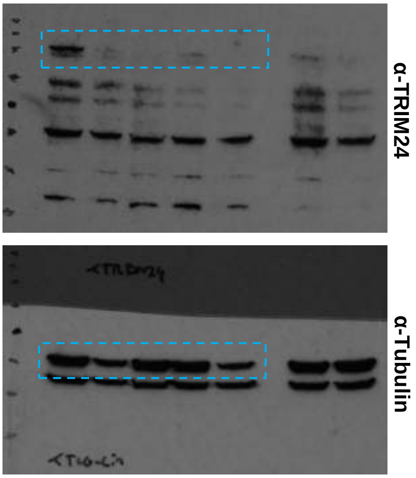

Fig. 5a

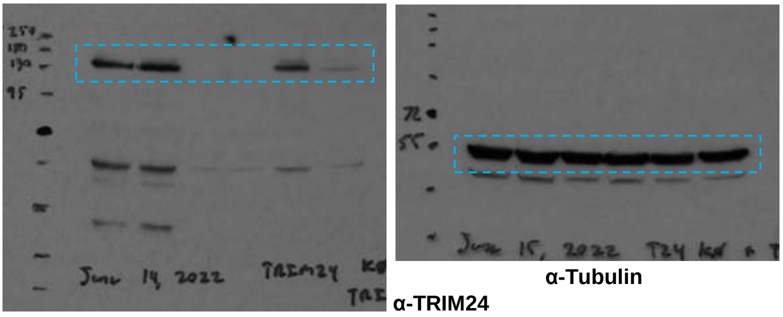

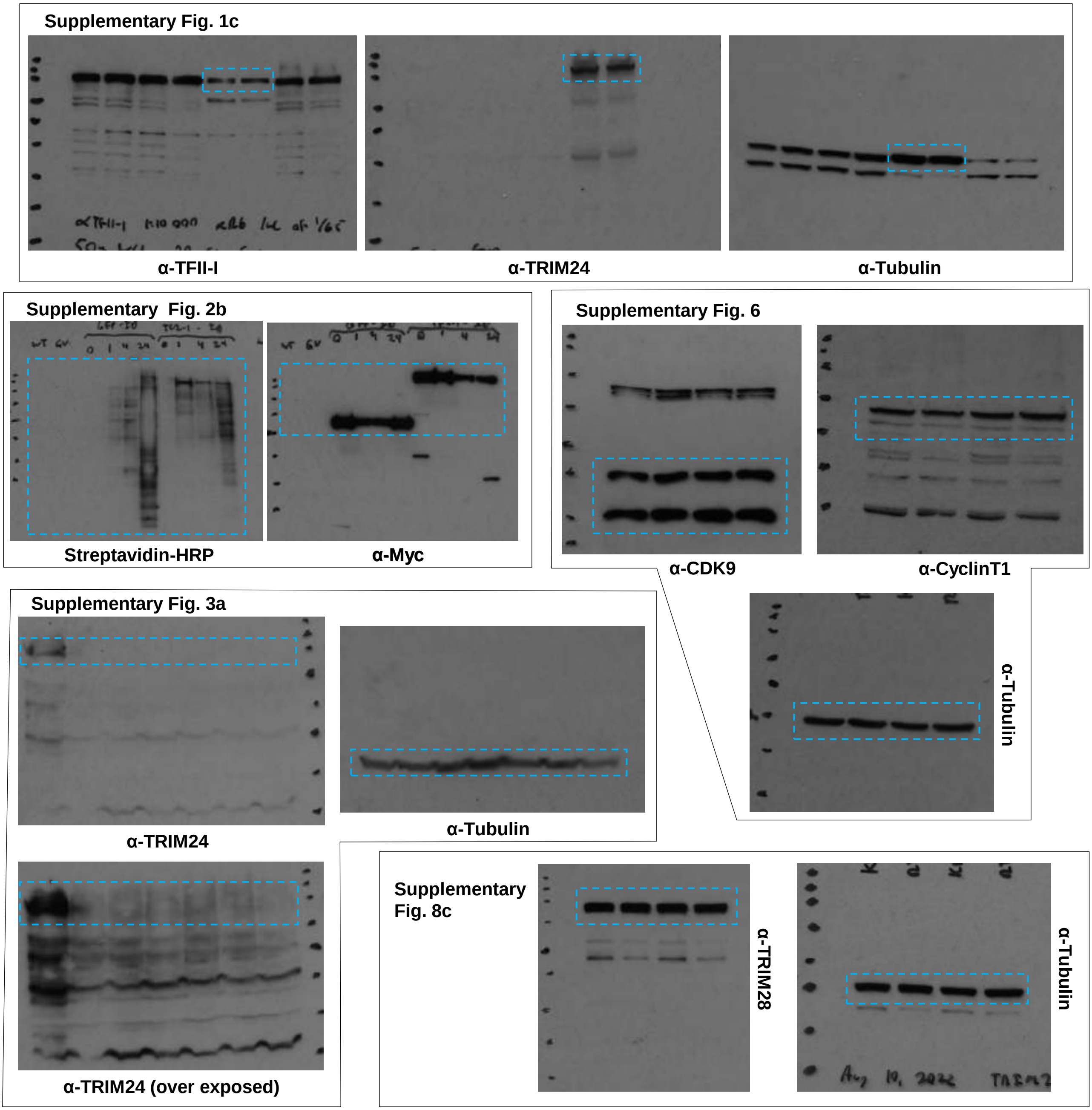
Supplementary Fig. 1c
α-TFII-I
α-TRIM24
α-Tubulin
Supplementary Fig. 2b
Streptavidin-HRP
α-Myc
Supplementary Fig. 6
α-CDK9
α-CyclinT1
α-Tubulin
Supplementary Fig. 3a
α-TRIM24
α-Tubulin
α-TRIM24 (over exposed)
Supplementary Fig. 8c
α-TRIM28
α-Tubulin

Supplementary Data 1. The source data graphs shown in the paper.

Supplementary Data 2. List of differentially expressed genes between wildtype and *TRIM24* KO, and control shRNA versus TFII-I shRNA transduced Jurkat mHIV-Luciferase T cells.

Supplementary Data 3. List of enriched biological process gene ontology terms from DAVID analysis.

Supplementary Data 4. List of differentially expressed genes between untreated and PMA/ ionomycin activated Jurkat mHIV-Luciferase T cell lines.
