# Supplementary material for: TRIM24 controls induction of latent HIV-1 by stimulating transcriptional elongation
Source: Commun Biol. 2023 Jan 23;6:86. doi: 10.1038/s42003-023-04484-z (PMC9870992; doi:10.1038/s42003-023-04484-z)
Supplement: Supplementary file 2 — Description of Additional Supplementary Data [file 42003_2023_4484_MOESM2_ESM.docx]

**Description of Additional Supplementary Files**

**File name:** Supplementary Data 1

**Description:** The source data behind the graphs in the paper

**File name:** Supplementary Data 2

**Description:** Differentially expressed genes between PMA/Ion stimulated wildtype and TRIM24 KO, and LKO versus TFII-I shRNA transduced Jurkat Tat cells. DESeq2 analysis, columns indicate: Gene ID; Mean normalized counts; Fold Change log2, FC log2; log2 FC standard error; Wald statistic; p-value; Adjusted p-value.

**File name:** Supplementary Data 3

**Description:** Biological process gene ontology (GO) terms identified from DAVID analysis of DEG genes. DEG from TRIM24 knockout or TFII-I shRNA transduced Jurkat cells as identified with DESeq2 were analyzed using Database for Annotation, Visualization and Integrated Discovery (DAVID).

**File name:** Supplementary Data 4

**Description:** Differentially expressed genes identified between untreated and PMA/ ionomycin activated Jurkat Tat cell lines. Tables detailing DEG between untreated and PMA/ ionomycin stimulated Jurkat T cells (LKO vector control), TRIM24 knockout or TFII-I shRNA transduced cells analyzed using DESeq2. Columns indicate: Gene ID; Mean normalized counts; Fold Change log2, FC log2; log2 FC standard error; Wald statistic; p-value; Adjusted p-value.
