# Supplementary material for: TRIM24 controls induction of latent HIV-1 by stimulating transcriptional elongation
Source: Commun Biol. 2023 Jan 23;6:86. doi: 10.1038/s42003-023-04484-z (PMC9870992; doi:10.1038/s42003-023-04484-z)
Supplement: Supplementary file 7 — Reporting Summary [file 42003_2023_4484_MOESM7_ESM.pdf]

nature portfolio

Corresponding author(s): Ivan Sadowski

Last updated by author(s): Dec 23, 2022

Reporting Summary

Nature Portfolio wishes to improve the reproducibility of the work that we publish. This form provides structure for consistency and transparency in reporting. For further information on Nature Portfolio policies, see our Editorial Policies and the Editorial Policy Checklist.

Statistics

For all statistical analyses, confirm that the following items are present in the figure legend, table legend, main text, or Methods section.

n/a	Confirmed	
☐	☒	The exact sample size (*n*) for each experimental group/condition, given as a discrete number and unit of measurement
☐	☒	A statement on whether measurements were taken from distinct samples or whether the same sample was measured repeatedly
☐	☒	The statistical test(s) used AND whether they are one- or two-sided<br>*Only common tests should be described solely by name; describe more complex techniques in the Methods section.*
☒	☐	A description of all covariates tested
☒	☐	A description of any assumptions or corrections, such as tests of normality and adjustment for multiple comparisons
☐	☒	A full description of the statistical parameters including central tendency (e.g. means) or other basic estimates (e.g. regression coefficient) AND variation (e.g. standard deviation) or associated estimates of uncertainty (e.g. confidence intervals)
☐	☒	For null hypothesis testing, the test statistic (e.g. *F*, *t*, *r*) with confidence intervals, effect sizes, degrees of freedom and *P* value noted<br>*Give P values as exact values whenever suitable.*
☒	☐	For Bayesian analysis, information on the choice of priors and Markov chain Monte Carlo settings
☒	☐	For hierarchical and complex designs, identification of the appropriate level for tests and full reporting of outcomes
☒	☐	Estimates of effect sizes (e.g. Cohen's *d*, Pearson's *r*), indicating how they were calculated

Our web collection on statistics for biologists contains articles on many of the points above.

Software and code

Policy information about availability of computer code

Data collection	N/A
Data analysis	GraphPad Prism 9.0.0 was used for statistical analysis.

For manuscripts utilizing custom algorithms or software that are central to the research but not yet described in published literature, software must be made available to editors and reviewers. We strongly encourage code deposition in a community repository (e.g. GitHub). See the Nature Portfolio guidelines for submitting code & software for further information.

Data

Policy information about availability of data

All manuscripts must include a data availability statement. This statement should provide the following information, where applicable:

- Accession codes, unique identifiers, or web links for publicly available datasets
- A description of any restrictions on data availability
- For clinical datasets or third party data, please ensure that the statement adheres to our policy

RNA-seq data has been submitted to GEO and is publicly available. The project title is "Effect of depletion of TFII-I or TRIM24 on Jurkat T cell activation" with the accession GSE221633. All other data pertaining to the findings of this study are available within the article and its Supplementary Information files, or are available from the authors upon request.

Human research participants

Policy information about studies involving human research participants and Sex and Gender in Research.

Reporting on sex and gender	NA
Population characteristics	NA
Recruitment	NA
Ethics oversight	NA

Note that full information on the approval of the study protocol must also be provided in the manuscript.

Field-specific reporting

Please select the one below that is the best fit for your research. If you are not sure, read the appropriate sections before making your selection.

☒ Life sciences ☐ Behavioural & social sciences ☐ Ecological, evolutionary & environmental sciences

For a reference copy of the document with all sections, see nature.com/documents/nr-reporting-summary-flat.pdf

Life sciences study design

All studies must disclose on these points even when the disclosure is negative.

Sample size	Sample size was determined in a manner that is consistent with previous literature using similar assays. For the majority of assays, measurements were taken in duplicate or triplicate.
Data exclusions	Two data points were excluded. As stated in "Statistics and reproducibility" these are in Fig. 3F, WT- EV and Fig. 7B, TRIM24 KO – Gag, both of which had one outlier value removed.
Replication	Biological replicates were performed as described in the Figure Legends.
Randomization	Randomization was not performed.
Blinding	Blinding was not performed as it was not relevant to this study.

Reporting for specific materials, systems and methods

We require information from authors about some types of materials, experimental systems and methods used in many studies. Here, indicate whether each material, system or method listed is relevant to your study. If you are not sure if a list item applies to your research, read the appropriate section before selecting a response.

Materials & experimental systems

n/a	Involved in the study	
☐	☒	Antibodies
☐	☒	Eukaryotic cell lines
☒	☐	Palaeontology and archaeology
☒	☐	Animals and other organisms
☒	☐	Clinical data
☒	☐	Dual use research of concern

Methods

n/a	Involved in the study	
☒	☐	ChIP-seq
☐	☒	Flow cytometry
☒	☐	MRI-based neuroimaging

Antibodies

Antibodies used	Antibodies used for western blotting are as follows: Tubulin - Abcam ab7291, TFII-I - Abcam ab134133, Flag - Sigma Aldrich F3165, Myc - Santa Cruz sc-40, TRIM24 - Proteintech 14208-1-AP, KAP1 - Proteintech 15202-1-AP, GAPDH - Abcam ab9484, CDK9 - Abcam ab239364, Cyclin T1 - Santa Cruz sc-10750, Streptavidin-HRP - Abcam ab7403, Goat Anti-Rabbit-HRP - Abcam ab6721, Goat Anti-Mouse-HRP - Pierce #1858413.<br><br>Antibodies used for immunoprecipitation, including ChIP-qPCR, are as follows: TFII-I - BD Biosciences 610842, TRIM24 - Proteintech

14208-1-AP, RNAPII - Abcam ab26721, RNAPII pS2 - Abcam ab238146, CDK9 - Abcam ab239364, NFκB p65 - Thermo Fisher 51-0500, KAP1 - Abcam ab10483, H3K27ac – Abcam ab4729, H3K9me3 – Abcam ab176916, Flag - Sigma Aldrich F3165, mouse IgG – Santa Cruz sc-2025.

Validation

All antibodies used are commercially available and have been validated by there respective manufacturer.

Eukaryotic cell lines

Policy information about cell lines and Sex and Gender in Research

Cell line source(s)	Jurkat E6-1, Jurkat Tat, and TZM-bl cells were obtained from NIH-AIDS reagent resource. HEK293T cells were obtained from ATCC.
Authentication	None of the cell lines have been authenticated following being obtained from the indicated supplier.
Mycoplasma contamination	Mycoplasma testing has been performed yearly with no positive tests.
Commonly misidentified lines (See ICLAC register)	Jurkat cells were used as they are a common immortalized human T cell line used to study HIV-1.

Flow Cytometry

Plots

Confirm that:

☒ The axis labels state the marker and fluorochrome used (e.g. CD4-FITC).

☒ The axis scales are clearly visible. Include numbers along axes only for bottom left plot of group (a 'group' is an analysis of identical markers).

☒ All plots are contour plots with outliers or pseudocolor plots.

☒ A numerical value for number of cells or percentage (with statistics) is provided.

Methodology

Sample preparation	Jurkat Tat TRIM24 TetOff cells were prepared as indicated in the text.
Instrument	BD Biosciences LSRII-561
Software	FlowJo (TreeStar) software was used for data analysis.
Cell population abundance	At least 10 000 gated, live cells were measured per sample.
Gating strategy	Forward scatter and side scatter thresholds were set so only live cells were counted. Uninfected Jurkat Tat TRIM24 TetOff cells were used to determine the fluorescent negative population which was gated in the bottom left quadrant shown in the flow cytometry scatter plots of Fig. 5C.

☒ Tick this box to confirm that a figure exemplifying the gating strategy is provided in the Supplementary Information.
